# Cancer-associated fibroblasts-derived CXCL12 enhances immune escape of bladder cancer through inhibiting P62-mediated autophagic degradation of PDL1

**DOI:** 10.1186/s13046-023-02900-0

**Published:** 2023-11-25

**Authors:** Zhao Zhang, Yongbo Yu, Zhilei Zhang, Dan Li, Zhijuan Liang, Liping Wang, Yuanbin Chen, Ye Liang, Haitao Niu

**Affiliations:** 1 Department of Urology, The Affiliated Hospital of Qingdao University, No.16 Jiangsu Road, Qingdao, 266000 China; 2 Key Laboratory, Department of Urology and Andrology, The Affiliated Hospital of Qingdao University, Qingdao, China; 3 Medicine College, Qingdao University, Qingdao, China; 4 Department of Urology, Weifang People’s Hospital, Weifang Medical University, Weifang, China

**Keywords:** Cancer-associated fibroblast, CXCL12, PDL1, Autophagy, P62, Immune escape

## Abstract

**Background:**

Cancer-associated fibroblasts (CAFs), the predominant stromal cell of tumor microenvironment (TME), play an important role in tumor progression and immunoregulation by remodeling extracellular matrix (ECM) and secreting cytokines. However, little is known about the details of the underlying mechanism in bladder cancer.

**Methods:**

Bioinformatics analysis was performed to analyze the prognostic value of CAFs and CXCL12 using GEO, TCGA and SRA databases. The effects of CXCL12 on bladder cancer progression were investigated through in vitro and in vivo assays. The biological mechanism of the effect of CXCL12 on PDL1 were investigated using western blotting, immunoprecipitation, RT-PCR, immunofluorescence, mass spectrometry, protein stability, and flow cytometry.

**Results:**

The results demonstrated that CAFs-derived CXCL12 promoted cancer cell migration and invasion and upregulated PDL1. Mechanistically, upon binding to its specific receptor, CXCL12 activated the downstream JAK2/STAT3 pathway and rapidly up-regulated the expression of deubiquitinase CYLD. CYLD deubiquitinated P62 causing P62 accumulation, which in turn inhibited the autophagic degradation of PDL1. In vivo experiments demonstrated that blocking CXCL12 inhibited tumor growth, reduced tumor PDL1 expression and increased immune cell infiltration.

**Conclusions:**

This study revealed a novel mechanism for the role of CXCL12 in P62-mediated PDL1 autophagic regulation. Combined application of CXCL12 receptor blocker and PD1/PDL1 blocker can more effectively inhibit PDL1 expression and enhance antitumor immune response. Targeting CAFs-derived CXCL12 may provide an effective strategy for immunotherapy in bladder cancer.

**Supplementary Information:**

The online version contains supplementary material available at 10.1186/s13046-023-02900-0.

## Introduction

Bladder cancer is the most common malignancy of the urinary system, with more than 570,000 new cases and over 200,000 deaths worldwide each year [1]. Approximately 75% of new cases are non-muscle-invasive bladder cancer (NMIBC) with a high rate of relapse and progression after transurethral resection [2]. The tumors that invade the detrusor muscle, called muscle-invasive bladder cancer (MIBC), account for about 25% of new diagnoses and have a high propensity to spread to surrounding organs and lymph nodes [3]. There are two treatment paradigms for MIBC. Radical cystectomy combined with neoadjuvant chemotherapy is a routine treatment option for non-bladder sparing. For patients who desire to retain their bladder, multi-modal regimen that includes maximal transurethral resection of bladder tumor (TURBT), chemotherapy and radiotherapy is another choice [4]. However, even after treatment, recurrence or metastasis occurs in up to 50% of patients. In addition, a large proportion of patients are considered unsuitable for platinum-based chemotherapy due to comorbidities [5]. In recent years, the emergence of immunotherapy has provided a new option for cancer patients. There are three generally recognized classes which include cancer vaccines (e.g. bacille Calmette-Guérin), tumor-targeting antibodies (e.g. enfortumab and sacituzumab) and immunomodulators (e.g. PD1/PDL1 inhibitor) [6]. And yet, due to individual heterogeneity of bladder cancer, some patients are not sensitive to immunotherapy [7]. Therefore, there is an urgent need to better understand the molecular mechanisms of bladder cancer to improve therapeutics.

The tumor behaviors and biological characteristics are dictated not only by tumor parenchyma but also by its tumor microenvironment (TME). The TME is complex and involves many different types of cells that can work together to drive complex and dynamic interactions between cancer cells and TME, thereby helping cancer cells evade immune surveillance and survive treatment [8]. Cancer-associated fibroblasts (CAFs), one of the major stromal cells in TME, are a highly heterogeneous population of stromal cells and an important component of solid tumors [9]. CAFs co-evolve with cancer cells by cell-cell interaction, cytokines secretion and extracellular matrix (ECM) remodeling. Thus, they can survive in the complex TME and contribute to tumor progression, angiogenesis, immune escape and drug resistance [10]. CAFs have also been reported to predict poor prognosis in many cancer types, including bladder cancer [11, 12]. Moreover, CAFs are emerging as an important player in shaping the immunosuppressive TME. They can inhibit the activity of effector and cytotoxic immune cells and increase the amount of regulatory T lymphocytes by secreting cytokines and upregulating immune checkpoint ligands [13]. Thus, targeting CAFs in bladder cancer may provide new insights into the field of cancer immunotherapy.

In the present study, we sought to explore the role of CAFs in bladder cancer prognosis and immunomodulation, and to identify which cytokine plays a key role. We then identified CXCL12 as the key cytokine and revealed a novel cytological mechanism by which CXCL12 regulates PDL1. Our study suggested that targeting CAFs-derived CXCL12 may provide an alternative approach to selectively inhibit PDL1 for promoting antitumor immunity.

## Materials and methods

### Bioinformatics analyses

RNA sequencing profile and clinical data of bladder cancer samples were downloaded from the TCGA data portal (http://cancergenome.nih.gov/). The public immunohistochemical information was obtained from the HPA portal (https://www.proteinatlas.org/). The cytokine gene list was obtained from the ImmPort database (https://www.immport.org/). The P62 interactor and deubiquitinases (DUBs) list was downloaded from the BioGRID database (https://thebiogrid.org/). The CAFs proportion of each sample was calculated by “EPIC”, “xCELL” and “MCP-counter” R package [14–16]. The “BLCAsubtyping” R packet was used to distinguish the molecular subtypes of the TCGA cohort [17]. Gene Set Enrichment Analysis (GSEA), Gene Ontology (GO) and Kyoto Encyclopedia of Genes and Genomes (KEGG) analysis were performed by “clusterProfiler” package [18]. Gene Set Variation Analysis (GSVA) was performed by “GSVA” R package [19]. “CIBERSORT” R package was used to analyze 22 types of immune cell fractions in each sample, with p > 0.05 were excluded [20]. Differentially expressed genes (DEGs) between groups were identified by “edgeR” R package.

The scRNA-seq dataset containing eight tumors was downloaded from the SRA portal (BioProject ID: PRJNA662018, https://www.ncbi.nlm.nih.gov/sra/) [21]. The raw data was processed by Cell Ranger (version 6.1) and converted into a Seurat object by the “Seurat” R package [22]. Seurat Object was filtered to exclude the cells that expressed fewer than 1000 genes, more than 6000 genes, greater than 10% mitochondrial genes, and more than 5% erythrocyte genes. “Harmony” R package was used to remove potential batch effect. To reduce the dimensionality of the scRNA-Seq dataset, principal component analysis (PCA) was performed. With Elbowplot function of Seurat, top 30 principal components (PC) were used to perform the downstream analysis. The main cell clusters were identified with the FindClusters function offered by Seurat, with resolution set as default (res = 0.8). And then they were visualized with UMAP plots. The Findallmarker function was performed to identify differentially expressed genes in clusters.

### Human specimens and patients

The Ethics Committee of the Affiliated Hospital of Qingdao University approved this study, and all participants provided informed consent (IRB number: QYFYWZLL28074). To obtain fibroblasts, fresh human tissue samples were collected from patients who received bladder resections. For immunohistochemistry, we collected tissue samples and clinical information from 56 patients diagnosed with bladder cancer at our hospital, 15 of whom had corresponding paracancerous tissue.

### Cell culture

The human bladder cancer cells T24, UMUC3 and human embryonic kidney cell 293T were purchased from the Cell Bank of the Chinese Academy of Sciences (Shanghai, China). The mouse bladder cancer cell MB49 was purchased from iCell Bioscience Inc (Shanghai, China). The cell lines were maintained in RPMI-1640 or DMEM medium supplemented with 10% fetal bovine serum (FBS; Gibco) at 37 °C under 5% CO2 in a standard humidified incubator. CAFs and normal fibroblasts (NFs) were obtained from fresh bladder cancer tissues and normal bladder tissues using the method described by Vivacqua [23]. In brief, tissues were cut into smaller pieces, placed in digestion solution (400 IU collagenase I, 100 IU hyaluronidase, and 10% FBS containing antibiotic and antimycotic solution) and incubated overnight at 37 °C. Cells were then separated by differential centrifugation and cultured in DMEM/F12 medium. The fibroblasts used in this study were less than 10 passages.

CD3 + T cells were obtained from blood samples of healthy volunteers using lymphocyte separation medium as described previously [24]. Briefly, 5 mL fresh peripheral blood was mixed with saline in a 1:1 ratio. The mixture was then slowly added to the liquid surface of 10 mL cell separation solution. The isolated peripheral blood mononuclear cells (PBMCs) were obtained after a series of layering, washing and centrifugation steps according to the manufacturer’s instruction. The PBMCs were cultured in a culture flask precoated with RetroNectin (T100A, Takara, Kusatsu, Japan) and anti-human CD3 antibody (T210, Takara, Kusatsu, Japan). IFN-γ (285-IF, Novus, Littleton, CO, USA) and interleukin-2 (200-02, PeproTech, Rocky Hill, NJ, USA) was added to activate and expand the CD3 + T cell.

### Antibodies and reagents

The antibodies and reagents used in this study were shown in Table S1.

### ELISA

CXCL12 concentration of fibroblast medium was analyzed using human SDF-1/CXCL12 ELISA Kit (E-EL-H0052c, Elabscience, Wuhan, China). All the assays were performed according to the protocols provided by the manufacturer.

### Transfection of plasmids or siRNAs

The human P62, CYLD, Ub, LC3 cDNAs were subcloned into pcDNA3.1(+) (Invitrogen Life technologies) and the plasmids were purchased from the Beijing Genomics Institute. The specific small interfering RNA (siRNA) of CXCR4, CXCL12, P62 and CYLD were purchased from GenePharma and the sequences are shown in Table S2. Cells were transfected with plasmids or siRNA using Lipofectamine 3000 and P3000 (L3000001, Thermo Fisher Scientific, Waltham, MA, USA) according to manufacturer’s instruction.

### Proliferation, migration and invasion assays

MTT (475989, Sigma-Aldrich, St. Louis, MO, USA) assay was used to detect cell proliferation. 2000 cells were inoculated in 96-well plates, each well containing 200 ml of complete growth medium, and cultured for the indicated times after treatment. Then the number of viable cells were measured by MTT methods. The Wound-Healing assay was used to detect the migration ability of cells. Scratch was applied with a 200 µl tip in a six-well plate and incubation was continued in FBS-free medium. Cells were photographed at 0 and 24 h after scratch. Migration rate was measured with the ImageJ software [25]. Cell invasion was performed by transwell chamber technology. Cells (5 × 10^4^) were inoculated in the upper chamber (Corning) with serum-free medium and the lower chamber was filled with medium containing 10% FBS. After 24 h, the cells on the membrane of transwell inserts were fixed with 4% paraformaldehyde and stained with 0.1% crystal violet.

### Western blotting (WB) and immunoprecipitation (IP)

Cell lysates were prepared using SDS lysis buffer. The BCA Protein Assay Kit (71285-M, ThermoFisher Scientific, Waltham, MA, USA) was used to quantify protein concentrations. For immunoprecipitation, cells were lysed with IP Lysis Buffer (20–188, ThermoFisher Scientific, Waltham, MA, USA) containing 1% cocktail of proteinase and phosphatase inhibitor and PMSF (S8830 and 329-98-6, ThermoFisher Scientific, Waltham, MA, USA). Equal amounts of protein lysate were incubated overnight at 4 °C with antibodies and protein A/G magnetic beads (HY-K0202, MedChemExpress, Monmouth Junction, NJ, USA). Then the beads were washed at least three times with lysis buffer, and proteins were eluted with 1×loading buffer for WB. Whole cell lysates or immunoprecipitated proteins were resolved by SDS-PAGE. The proteins in the gel were transferred to PVDF membranes.

### LC-MS/MS analysis

Briefly, we used a scraper to harvest T24 cells and lysed the cells with IP lysis buffer. Then, we carried out SDS-PAGE and placed the gel into Coomassie brilliant blue solution for staining. Next, we collected the protein bands and used LC-MS/MS for analysis. The peptides were separated by a Thermo Scientific EASY-nLC 1000 system. The isolated peptide was detected by a Q-Exactive mass spectrometer (Thermo Scientific). The raw data was processed by Mascot software by searching human UniProt database.

### Quantitative real time RT-PCR

Total RNA was extracted using AG RNAex Pro Reagent and cDNA was synthesized with the Evo M-MLV RT Mix Kit (AG11728, Accurate Biotechnology, Hunan, China). qPCR was performed with the Roche LightCycler 480II real-time PCR detection system (Roche, Basel, Switzerland). Expression level was normalized to that of β-actin. The primers for qRT-PCR are listed in Table S2.

### Immunofluorescence

Cells grown on cell slide were fixed with 4% paraformaldehyde, permeabilized with 0.5% Triton X-100, blocked with 5% BSA, and incubated with primary antibody overnight at 4 °C, followed by fluorescent-dyeconjugated secondary antibodies. DAPI was used to stain the nuclei. A laser scanning confocal microscope was used to photograph immunofluorescence expression.

### Immunohistochemistry (IHC)

IHC was performed as described previously [12]. Paraffin-embedded samples were cut into 4-µm sections and immunostained with primary antibodies. Sections were imaged with an optical microscope (Olympus), and staining intensity was assessed according to the semi-quantitative scoring system in considering the staining intensity and area extent. Two independent pathologists scored the sections (double-blinded). The staining intensity was scored as 0 (negative), 1 (weak), 2 (moderate), and 3 (strong). The staining extent was scored as 0 (0%), 1 (1–25%), 2 (26–50%), 3 (51–75%), and 4 (76–100%) according to the percentage of positively stained cells. The final IHC score was determined by multiplying the two scores, yielding a range from 0 to 12.

### Flow cytometry

Collect 2 × 10^6^ cells from single cell suspension and resuspend pellet in 50 µl PBS containing Fc blocking and live/dead stain. Then add antibodies mix for surface or intracellular (need fixation and permeabilization) staining and incubate at 4 °C for 30 min. Flow cytometry was performed using standard protocol on CytoFLEX LX analyzer (Beckman Coulter) and analyzed with FlowJo software. The Annexin V-FITC/PI Apoptosis Detection Kit was purchased from Yeasen Biotechnology (Cat#40302). The antibodies used for flow cytometry were shown in Table S1.

### Animal studies

All animal experiments were conducted in accordance with the ethical obligations and animal care guidelines approved by Qingdao University. 5-week-old female C57BL/6 mice were housed in in a standardized animal laboratory. MB49 bladder tumors were established by subcutaneously injecting cells (1.5 × 10^6^ cells in 50% matrigel in PBS, 100 µL/mouse). Five days after tumor cell implantation, mice were randomly divided into designated experimental groups (n = 6 for each group) with comparable average tumor size and received different treatments. AMD3100 or BMS-1 was administered intraperitoneally every other day dosing at 5 mg/kg. Tumors were evaluated every 2 days by measuring the length and width of with a vernier caliper. Tumor volume (mm^3^) = 1/2 × (length) × (width)^2^. After the sacrifice of mice, tumors were weighed and recorded.

### Statistical analysis

Student’s t-test was used to compare the independent samples between two groups and one-way ANOVA was used when more than two groups. Wilcoxon rank-sum test or Kruskal-Wallis test was for groups without normal distribution. ImageJ was used to quantify the western blotting and immunofluorescence results. All experimental data are expressed as the mean ± standard deviation (S.D.) of at least three replicate experiments. P value below 0.05 was considered of statistical significance.

## Result

### CAFs infiltration was strongly associated with prognosis and immunomodulation of Bladder cancer

**Fig. 1:**
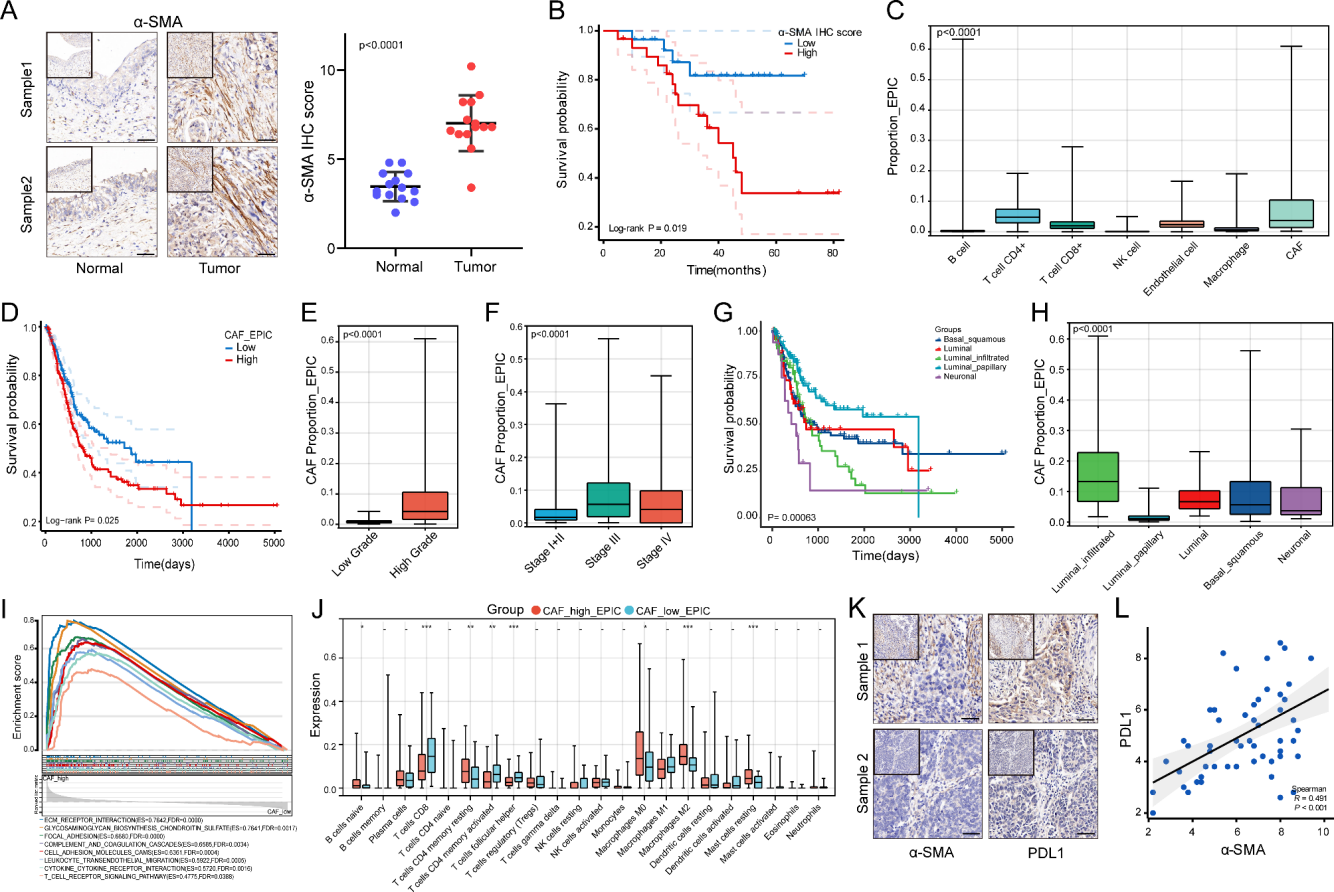
Correlation of CAFs infiltration with clinical features and immunomodulation in bladder cancer. (**A**) Left: Immunohistochemistry of α-SMA in bladder cancer tissues and adjacent normal tissues. Scale bar = 50 μm. Right: Quantitative IHC analysis of α-SMA staining (n = 15). (**B**) Comparison of overall survival between high and low IHC score groups of α-SMA in bladder cancer tissues (n = 56). (**C**) Proportion of various stromal cells calculated by EPIC algorithm of TCGA data. (**D**) Comparison of overall survival between high and low CAFs proportion groups of TCGA data (n = 406). (**E**, **F**) Correlation of CAFs proportion with tumor grade and clinical stage of TCGA data. (**G**) Comparison of overall survival for five molecular subtypes of TCGA data (n = 406). (**H**) Comparison of CAFs proportion among five molecular subtypes. (**I**, **J**) GSEA and CIBERSORT immune infiltration analysis between high and low CAFs groups in TCGA. (**K**) IHC results showed that PDL1 was highly expressed in the region of high α-SMA expression. Scale bar = 50 μm. (**L**) Correlation of IHC scores of α-SMA with IHC scores of PDL1. * P < 0.05; ** P < 0.01; *** P < 0.001

α-SMA is a classic marker gene for the identification of CAFs in the tumor microenvironment [26]. Our clinical samples were consistent with the IHC results from the HPA database, in that α-SMA expression was significantly higher in tumor tissues than in normal tissues (Fig. 1A, Figure S1A). The IHC results showed that patients with high α-SMA expression had a worse prognosis, which was consistent with the TCGA database result (Fig. 1B, Figure S1B). The data of bladder cancer in TCGA database showed that CAFs accounted for a high proportion in many stromal cells. (Fig. 1C). Patients with a higher CAFs percentage had a worse prognosis, a result confirmed by two other CAFs calculation algorithms and other GEO datasets (Fig. 1D, Figure S1C-F). In addition, the proportion of CAFs was higher in patients with high pathologic grade or high clinical stage (Fig. 1E, F).

Patients in the TCGA database were classified into five molecular subtypes: basal-squamous, luminal, luminal-infiltrated, luminal-papillary and neuronal (Fig. 1G). We found that the luminal-papillary type, which has the best prognosis, has the lowest percentage of CAFs. Luminal-infiltrated type was reported to be sensitive to immunotherapy, while it has the highest percentage of CAFs, which aroused our interest (Fig. 1H). The GSEA results showed a significant enrichment of multiple pathways related to the TME and immune regulation in the high proportion CAFs group (Fig. 1I). CIBERSORT analysis showed that CD8 T cells and activated CD4 T cells were significantly reduced in the high CAFs group (Fig. 1J). PDL1 is the most common immune checkpoint gene, and our IHC results showed a significant positive correlation between α-SMA and PDL1, suggesting that CAFs may regulate PDL1 expression (Fig. 1K, L).

### Identification of CXCL12 as a key CAFs-derived cytokine with prognostic value

**Fig. 2:**
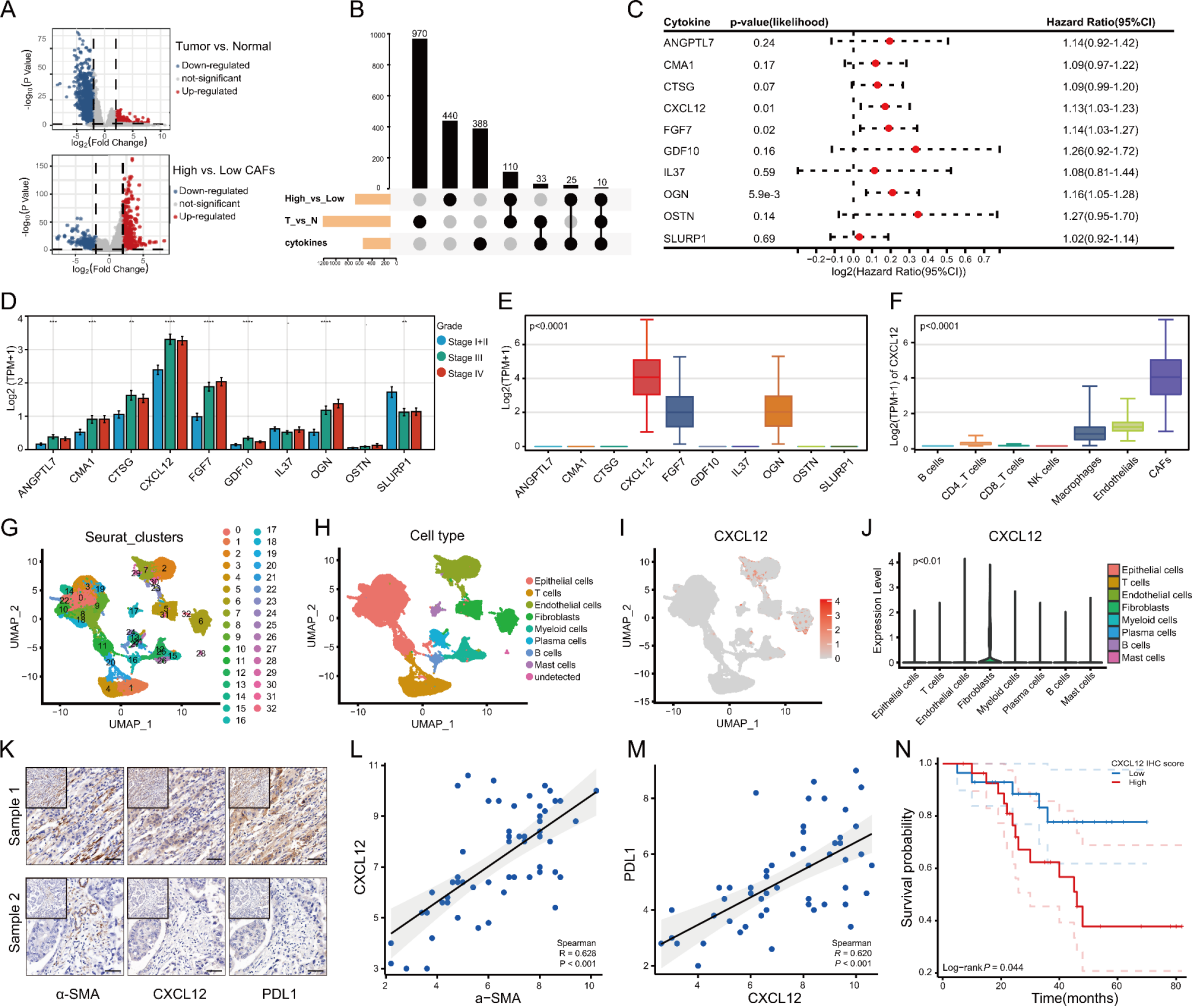
Identification of CXCL12 as a key CAF-derived cytokine in bladder cancer. (**A**) The DEGs between tumor and normal tissues in TCGA (up), and the DEGs between high and low CAFs proportion groups in TCGA (down). (**B**) Venn plot of the DEGs and cytokine list. (**C**) Forest plot for univariate Cox analysis of 10 cytokines. (**D**) Comparison of expression levels of 10 cytokines at different clinical stages. (**E**) Comparison the expression levels of 10 cytokines in CAFs (EPIC algorithm) of TCGA data. (**F**) Comparison of CXCL12 expression levels in different cell types of EPIC algorithm. (**G**) UMAP plot of Seurat clusters for single cells. (**H**) UMAP plot of single cells profiled by major cell types. (**I**, **J**) Comparison of the distribution and expression of CXCL12 in different single cell types. (**K**) IHC results showed that CXCL12 and PDL1 were highly expressed in the α-SMA high-expression region and vice versa. (**L**) Correlation of IHC scores of α-SMA with IHC scores of CXCL12. (**M**) Correlation of IHC scores of CXCL12 with IHC scores of PDL1. (**N**) Comparison of overall survival between high and low IHC score groups of CXCL12 in bladder cancer tissues (n = 56). ** P < 0.01; *** P < 0.001; **** P < 0.0001

CAFs are the main producers of ECM components and supports tumor cells by secreting various factors [27]. Next, we explored which secreted factor plays a key role. 10 cytokines were obtained by taking the intersection of the DEGs between the high and low proportion CAFs groups, the DEGs between tumor and normal tissue, and the cytokine list from the Immport database (Fig. 2A, B). The univariate Cox result showed that CXCL12, FGF7 and OGN were the risk factors for overall survival (Fig. 2C). Among them, CXCL12 was associated with both prognosis and clinical stage and had the highest expression (Fig. 2D, E). TCGA data showed the highest expression of CXCL12 in CAFs than other cell types by applying EPIC algorithm (Fig. 2F). Then the bladder cancer scRNA-seq data downloaded from the article by Chen et al. was analyzed [21]. After quality control and processing of the data, 80,585 single cells were clustered into eight major clusters (Fig. 2G, H; Figure S1G). The result showed that CXCL12 was expressed in CAFs and in some endothelial cells, and was significantly highly expressed in CAFs (Fig. 2I, J). Our IHC results and the TCGA database corroborated this finding, with CXCL12 also highly expressed in regions of high α-SMA expression (Fig. 2K, L; Figure S2H). Interestingly, CXCL12 also showed a significant positive correlation with PDL1 and was associated with poor prognosis in multiple datasets (Fig. 2M, N; Figure S1I-K).

### CAFs-derived CXCL12 promoted Bladder cancer growth and regulated PDL1 expression in vivo and in vitro

**Fig. 3:**
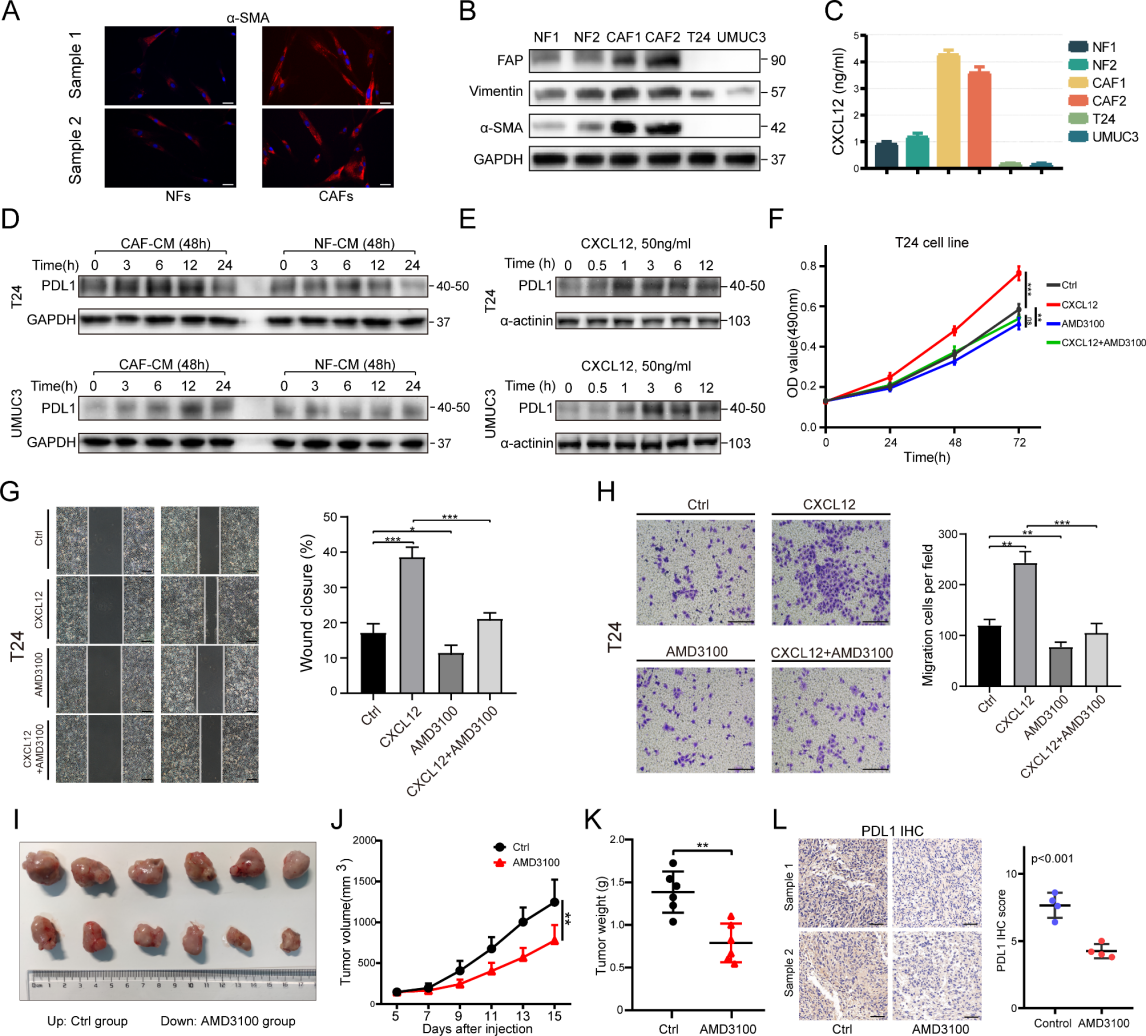
Extraction of CAFs / NFs and the promoting effect of CXCL12 on tumor. (**A**) Immunofluorescence of α-SMA expression in CAFs and NFs. Scale bar = 50 μm. (**B**) Western blotting showing the identification of CAFs and NFs. α-SMA and FAP were marker genes for CAFs. Vimentin was marker gene for mesenchymal cells. (**C**) Detection of CXCL12 secreted levels in culture medium (CM) of NFs, CAFs and bladder cancer cells after 48 h of culture using ELISA kit. (**D**) T24 cells and UMUC3 cells were treated with CAF or NF culture medium (CM) for 0–24 h, detected by western blotting with PDL1. (**E**) T24 cells and UMUC3 cells were treated with CXCL12 for 0–12 h, detected by western blotting with PDL1. (**F**) After the T24 cells had been treated with PBS, CXCL12 (50 ng/ml), AMD3100 (10 µg/ml), or AMD3100 + CXCL12 for 24, 48 and 72 h, the cell viability was assessed using MTT assay. (**G**) Wound-healing assay and (**H**) transwell invasion assay were performed following treatment with PBS, CXCL12 (50 ng/ml), AMD3100 (10 µg/ml), or AMD3100 + CXCL12 for 24 h in T24 cells. The statistical results were from three independent experiments. Values are means with SD. Scale bar = 300 μm. (**I**) The images of resected tumors in control and AMD3100 groups. (**J**) The tumor growth curve of each group. Data were presented as means ± SD (n = 6). (**K**) The tumor weight of each group. Data were presented as means ± SD (n = 6). (**L**) Left: PDL1 expression in IHC of control and AMD3100 groups. Right: Quantitative IHC analysis of PDL1 staining (n = 4)

We first extracted and identified CAFs and NFs. The results showed significantly high expression of α-SMA and FAP in CAFs (Fig. 3A, B). ELISA results of culture medium (CM) showed that CAFs secreted CXCL12 at the highest level, followed by NF at a lower level, while bladder cancer cells only secreted trace amounts (Fig. 3C). CAF1 and NF1 were used for subsequent research. Intervention of bladder cancer cells with CAF-CM or NF-CM revealed that CAFs could promote a more significant upregulation of PDL1 (Fig. 3D). In addition, under the stimulation of recombinant human CXCL12, PDL1 also showed a trend of rapid up-regulation with increasing time (Fig. 3E; Figure S2A).

The results of MTT assay, Wound-healing assay and transwell invasion assay showed that CXCL12 significantly promoted the proliferation, migration and invasion abilities of bladder cancer, and its antagonist AMD3100 was effective in reversing this supportive effect (Fig. 3F-H; Figure S2B-D). At last, to investigate the effect of blocking CXCL12 on tumor growth in vivo, we established tumor models. The treatment group received peritoneal injection of AMD3100 (5 mg/kg) every two days starting from the 5th day after tumor cell inoculation. The control group was given an equal amount of saline with peritoneal injection. The results showed that AMD3100 was able to inhibit tumor growth and reduce PDL1 expression (Fig. 3I-L). Collectively, these data suggested that CXCL12 had a strong tumor-promoting effect and could affecting PDL1 expression.

### CXCL12 inhibited autophagic degradation of PDL1

**Fig. 4:**
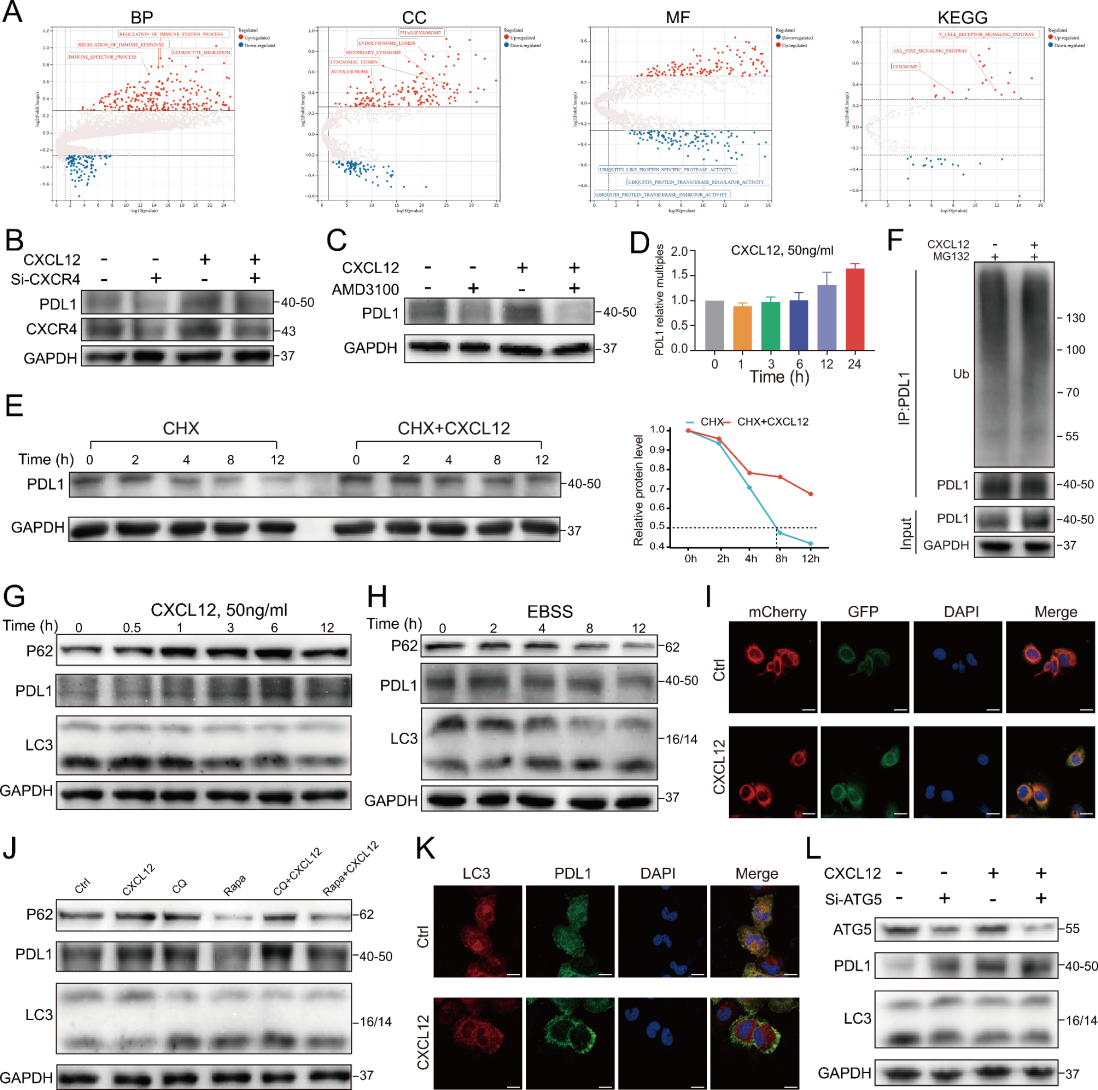
Inhibition of autophagy degradation of PDL1 by CXCL12. (**A**) Differential expression pathways targeting GO and KEGG of GSVA analysis between high and low CXCL12 expression groups in TCGA. BP: biological process. CC: cellular component. MF: molecular function. (**B**, **C**) The effect of CXCL12 (50 ng/ml) on PDL1 expression with or without siCXCR4 or AMD3100 (10 µg/ml), detected by western blotting in T24 cells. (**D**) The expression of PDL1 within 0–24 h after CXCL12 stimulation detected by qPCR in T24 cells. (**E**) Left: The degradation rate of PDL1 detected at the indicated time points by CHX chase assay (20µM) in T24 cells treated with or without CXCL12 (50 ng/ml) stimulation. Right: Quantitative curve of the level of remained PDL1. (**F**) Western blotting of PDL1 ubiquitination stimulated by CXCL12 in T24 cells. (**G**) Detection of the effect of CXCL12 on autophagy and PDL1 in T24 cells by western blotting with PDL1, P62 and LC3. (**H**) After inducing autophagy in T24 cells using EBSS, the expression of PDL1 was detected by western blotting. (**I**) Immunofluorescence showing the T24 cells that transfected EGFP-mCherry-LC3 plasmid co-incubated with CXCL12 (50ng/ml, 6 h). Scale bars = 20 μm. (**J**) The effect of chloroquine (10 µM, 12 h) or rapamycin (1 µM, 12 h) on PDL1 expression and autophagy with or without CXCL12 (50ng/ml) stimulation detected by western blotting in T24 cells. (**K**) Immunofluorescence showing the colocalization between PDL1 and LC3 after CXCL12 stimulation (10 µM, 12 h) in T24 cells. (**L**) Western blotting showing the effect of knocking down ATG5 on PDL1 with or without CXCL12 (50 ng/ml, 6 h) stimulation in T24 cells

GSVA analysis between high and low CXCL12 groups in TCGA database showed differences in multiple pathways related to immunomodulation, autophagy and ubiquitin (Fig. 4A). To confirm the effect of CXCL12 on PDL1, we found that PDL1 was downregulated after knocking down its receptor CXCR4 or applying the antagonist AMD3100, and there was no significant up-regulation of PDL1 after re-addition of CXCL12 (Fig. 4B, C; Figure S3A, B). Further studies found that CXCL12 had no effect on PDL1 mRNA within 12 h, but inhibited the rate of PDL1 degradation (Fig. 4D, E; Figure S3C, D). Protein degradation mainly occurs through two pathways, ubiquitin-proteasome system and autophagy-lysosome pathway. Next, we explored the impact of CXCL12 on these two pathways of PDL1.

After application of the proteasome inhibitor MG132, CXCL12 had no significant effect on the level of ubiquitination of PDL1, implying that CXCL12 did not affect this pathway (Fig. 4F; Figure S3E). Interestingly, CXCL12 showed a tendency to inhibit autophagy while promoting PDL1 elevation, as evidenced by the P62 promotion and reduction of LC3-II (Fig. 4G; Figure S3F). After EBSS induced autophagy, PDL1 was also downregulated (Fig. 4H; Figure S3G). In addition, EGFP-mCherry-LC3 plasmid was transfected into bladder cancer cells to visualize the effect of CXCL12 on autophagy. The green fluorescence of EGFP will be quenched under the acidic condition when autophagosomes fuse with lysosomes, but red mCherry can exist stably. Yellow puncta are usually regarded as autophagosomes (GFP + mCherry+). When autophagy flux is blocked, the number of yellow puncta in merged image increases, manifested as a decrease or no change in red signals and an increase in green signals. We found that CXCL12 induced an increase of both yellow and green puncta, which suggested the downregulation of autophagy flux (Fig. 4I; Figure S3H). Moreover, the autophagy inhibitor chloroquine (CQ) could also upregulate PDL1. The autophagy activator rapamycin (Rapa) downregulated PDL1 upon activation of autophagy. Application of Rapa followed by addition of CXCL12 inhibited the trend toward autophagy activation and brought back PDL1 (Fig. 4J; Figure S3I). The IF results also showed that CXCL12 resulted in an obvious reduction of the co-localized region of LC3 with PDL1 (Fig. 4K; Figure S3J). Finally, when autophagy initiation gene ATG5 was knocked down, we found that PDL1 showed significant upregulation. If CXCL12 stimulation was continued after silencing ATG5, there would be no more upregulation of PDL1 (Fig. 4L; Figure S3K). These findings indicated that CXCL12 reduced PDL1 degradation by inhibiting autophagy flux.

### P62 mediated autophagic regulation of PDL1 by CXCL12

**Fig. 5:**
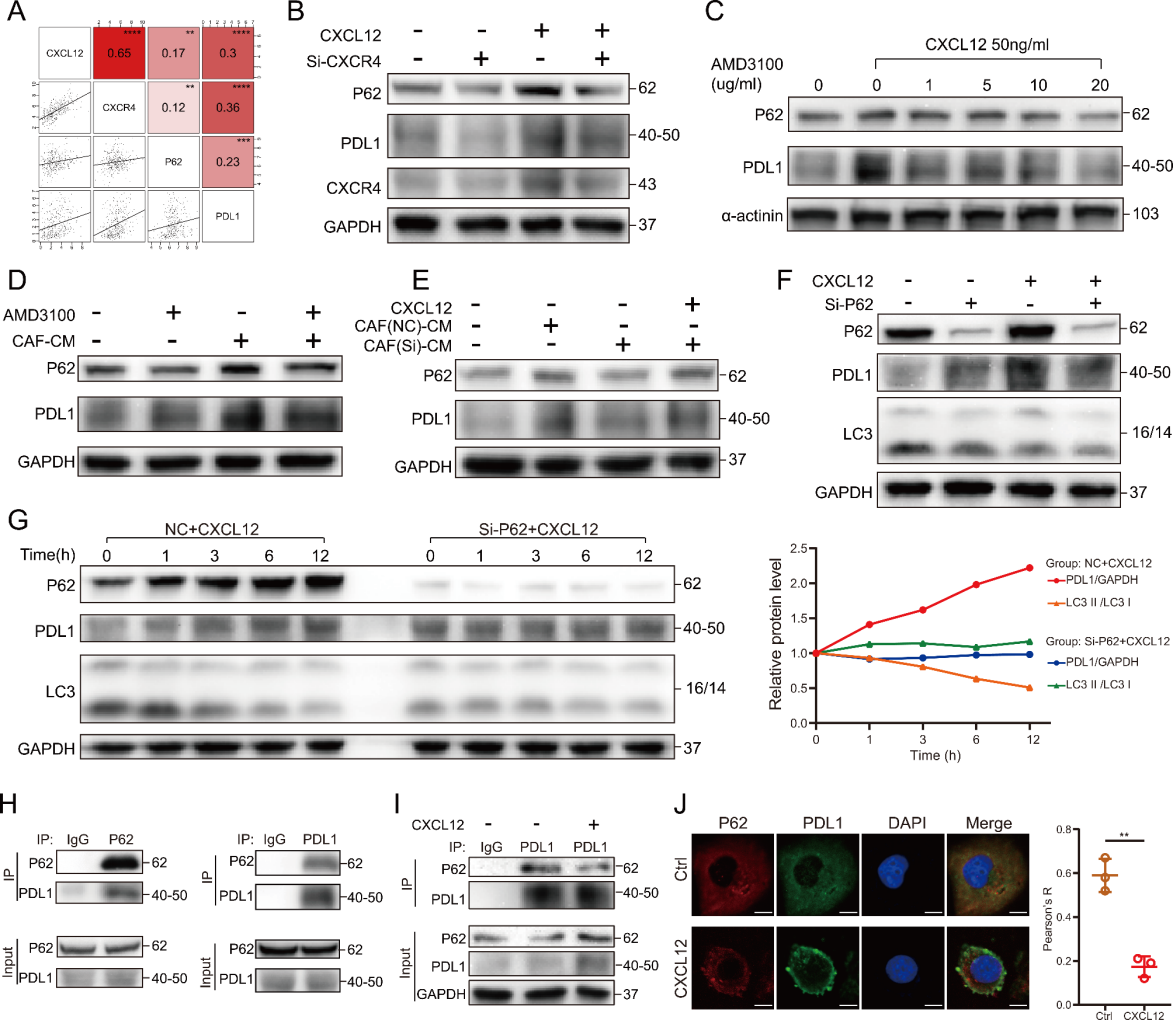
P62 mediated autophagic regulation of PDL1 by CXCL12. (**A**) Correlation between CXCL12, CXCR4, P62 and PDL1 in TCGA bladder cancer data. The coordinate axis represents log_2_ (gene expression). (**B**) Western blotting showing the effect of knocking down CXCR4 on P62 and PDL1 with or without CXCL12 (50 ng/ml, 6 h) stimulation in T24 cells. (**C**) The effect of CXCL12 (50 ng/ml, 6 h) stimulation on P62 and PDL1 expression was examined by western blotting after pretreatment of T24 cells using different concentrations of AMD3100. (**D**) Western blotting showing the effect of CAF-CM (12 h) on P62 and PDL in T24 cells with or without AMD3100 pretreatment (10ug/ml,12 h). (**E**) After knockdown of CXCL12 in CAFs, its culture medium (CM, 12 h) was used to stimulate T24 cells and then CXCL12 (50 ng/ml, 6 h) was added to rescue. Western blotting was used to detect changes of P62 and PDL1. (**F**) Western blotting showing the effect of knocking down P62 on PDL1 with or without CXCL12 (50 ng/ml, 6 h) stimulation in T24 cells. (**G**) Left: Compare the effect of CXCL12 (50 ng/ml, 0-12 h) on PDL1 and LC3 between the P62 knockdown and control groups of T24 cells by western blotting. Right: Quantitative curves of protein changes of PDL1 and LC3. (**H**) Co-immunoprecipitation (co-IP) showing the physical interaction between PDL1 and P62 in T24 cells. (**I**) Co-IP showing the effect of CXCL12 (50 ng/ml, 6 h) on the interaction between PDL1 and P62 in T24 cells. (**J**) Left: Immunofluorescence showing the colocalization between PDL1 and P62 after stimulation with CXCL12 (50 ng/ml, 6 h) in T24 cells. Scale bar = 10 μm. Right: The statistical results of colocalization (Pearson’s R value). Values are means ± SD from three independent experiments. ** P < 0.01; *** P < 0.001; **** P < 0.0001

P62 is a multidomain protein that acts as an autophagy receptor to mediate selective autophagy [28]. In the previous results, we found that P62 and PDL1 always had the same trend. TCGA data also showed a positive correlation between CXCL12/CXCR4, P62, and PDL1 (Fig. 5A). We therefore speculated whether P62 was directly involved in the regulation of PDL1. Knocking down CXCR4 or applying the antagonist AMD3100 can effectively counteract the promotion of CXCL12 on P62 and PDL1 (Fig. 5B, C; Figure S3A, B). Moreover, the CAF-CM could upregulate P62 and PDL1 simultaneously, and AMD3100 inhibited this promoting effect (Fig. 5D; Figure S4B). If the CXCL12 gene of CAFs was knocked down (Figure S4A), the promotion effect of CAF-CM on P62 and PDL1 was significantly inhibited. When exogenous CXCL12 was added, P62 and PDL1 increased again (Fig. 5E; Figure S4C).

Next, we found an interesting phenomenon that when P62 was knocked down, PDL1 was elevated instead. When CXCL12 was added again, PDL1 was not significantly up-regulated compared to P62 knockdown (Fig. 5F; Figure S4D). To clarify this phenomenon, we observed the effect of CXCL12 on PDL1 and autophagy over time after knocking down P62. The results showed that CXCL12 did not gradually promote the elevation of PDL1 and had essentially no effect on LC3 after knockdown of P62 compared to the control (Fig. 5G; Figure S4E). In addition, the IP results confirmed the existence of protein interaction between PDL1 and P62 (Fig. 5H; Figure S4F). Although the expression of both P62 and PDL1 increased after CXCL12 stimulation, the binding between the two decreased (Fig. 5I; Figure S4G). The results of immunofluorescence also showed a significant reduction in the co-localization of P62 with PDL1 after CXCL12 intervention (Fig. 5J; Figure S4H).

### CXCL12 reduced P62 ubiquitination

**Fig. 6:**
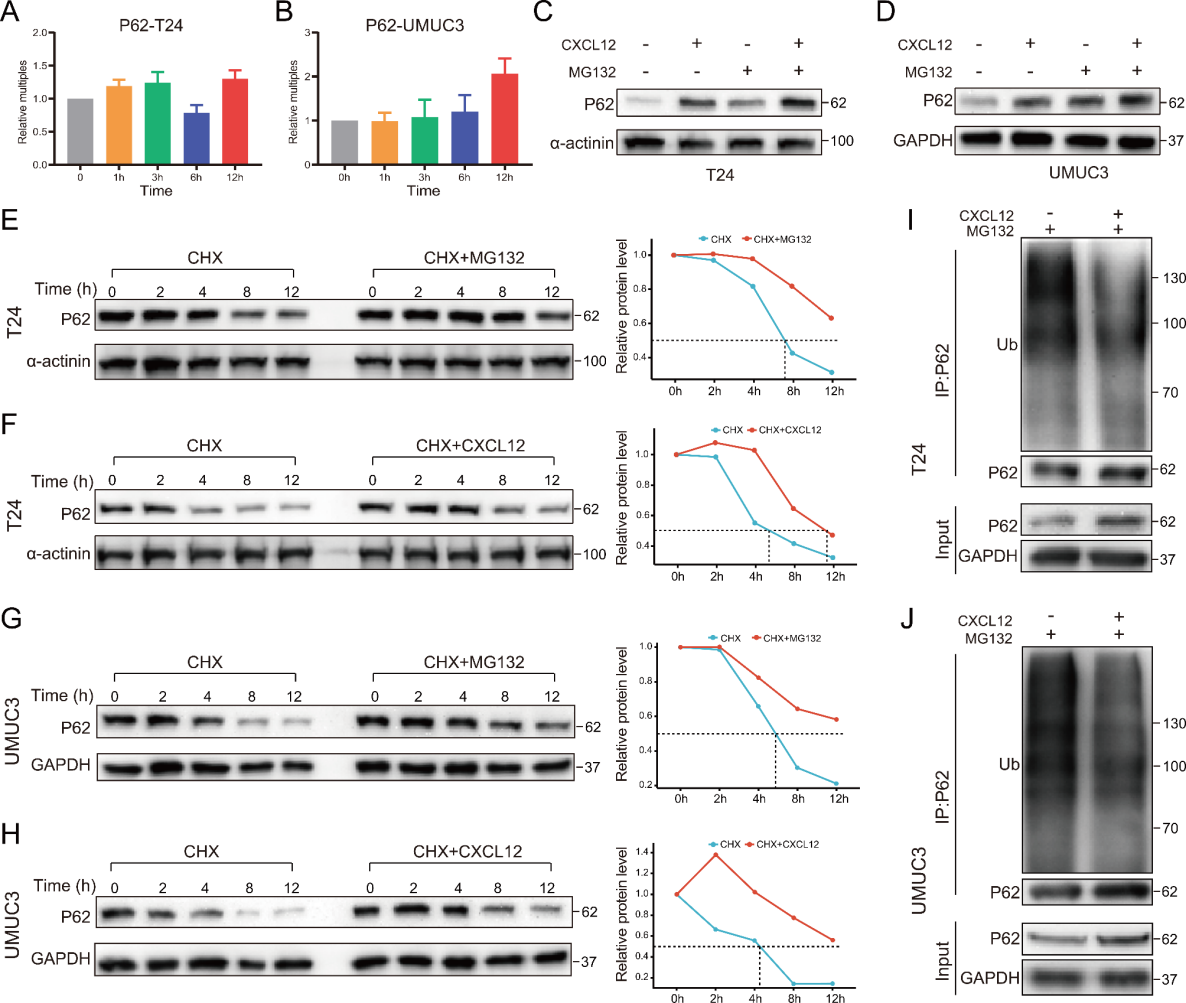
CXCL12 mediated the reduction of P62 ubiquitination. (**A**, **B**) The expression of P62 within 0–12 h after CXCL12 stimulation detected by qPCR in T24 and UMUC3 cells. (**C**, **D**) Western blotting showing the effect of MG132 (10µM, 6 h) and CXCL12 (50 ng/ml, 6 h) stimulation on P62 in T24 and UMUC3 cells. (**E**, **F**) Left: The degradation rate of P62 detected at the indicated time points by CHX chase assay (20µM) in T24 cells treated with MG132 (**E**) or CXCL12 (**F**) stimulation. Right: Quantitative curve of the level of remained P62. (**G**, **H**) Left: The degradation rate of P62 detected at the indicated time points by CHX chase assay (20µM) in UMUC3 cells treated with MG132 (**G**) or CXCL12 (**H**) stimulation. Right: Quantitative curve of the level of remained P62. (**I**, **J**) Western blotting of P62 ubiquitination stimulated by CXCL12 in T24 and UMUC3 cells

In our previous results, we observed that the change rate of P62 after stimulation by CXCL12 was more rapid than that of PDL1 and LC3. However, the RNA level of P62 was not significantly upregulated in a short period (Fig. 6A, B). We conjectured that CXCL12 was able to affect a certain protein degradation pathway of P62. Many studies have demonstrated the presence of ubiquitination of P62, which had been shown to play a key role in the regulation of autophagy [29]. The cells were first treated with the proteasome inhibitor MG132 to confirm that P62 had an obvious ubiquitin-proteasome degradation pathway in two cell lines of bladder cancer (Fig. 6E, G). CXCL12 stimulation could also significantly prolong the degradation half-life of P62 (Fig. 6F, H). When combined with CXCL12 on the basis of MG132, the trend of elevated P62 became more pronounced (Fig. 6C, D). The IP results showed that CXCL12 significantly inhibited the ubiquitination of P62, thereby maintaining P62 protein stability (Fig. 6I, J).

### CXCL12 promoted CYLD transcription and expression to maintain P62 protein stability

**Fig. 7:**
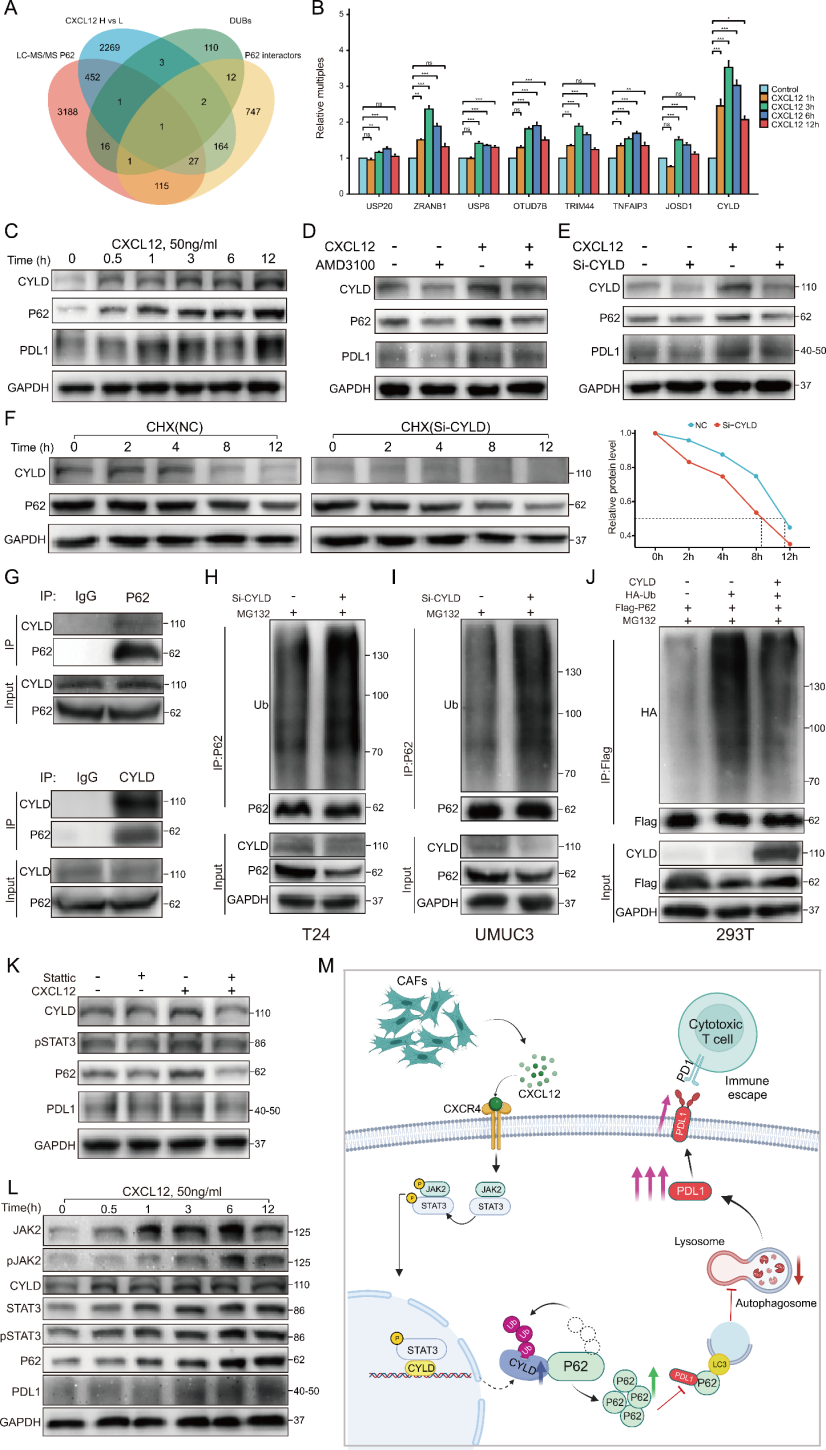
CYLD was regulated by CXCL12 and could deubiquitinate P62. (**A**) Venn plot for gene sets including CXCL12 H vs. L DEGs (upregulation), LC-MS/MS P62 result, deubiquitinases (DUBs) list and P62 interactors. (**B**) The expression of eight DUBs within 0–12 h after CXCL12 (50 ng/ml) stimulation detected by qPCR in T24 cells. (**C**) T24 cells were treated with CXCL12 for 0–12 h, detected by western blotting with CYLD, P62 and PDL1. (**D**) Western blotting showing the effect of CXCL12 (50ng/ml, 6 h) on CYLD, P62 and PDL1 in T24 cells with or without AMD3100 pretreatment (10ug/ml,12 h). (**E**) Western blotting showing the effect of knocking down CYLD on P62 and PDL1 with or without CXCL12 (50ng/ml, 6 h) stimulation in T24 cells. (**F**) Left: The degradation rate of P62 detected at the indicated time points by CHX chase assay (20µM) in T24 cells interfered by siCYLD. Right: Quantitative curve of the level of remained P62. (**G**) Co-immunoprecipitation (co-IP) showing the physical interaction between CYLD and P62 in T24 cells. (**H**, **I**) Western blotting showing the effect of knocking down CYLD on P62 ubiquitination in T24 and UMUC3 cells. (**J**) 293T cells transfected with the indicated plasmids for exogenous validation of the effect of CYLD on P62. (**K**) Western blotting showing the effect of CXCL12(50 ng/ml, 6 h) on CYLD, P62 and PDL1 with or without Stattic (5µM, 12 h) pretreatment in T24 cells. (**L**) T24 cells were treated with CXCL12 for 0–12 h, detected by western blotting with JAK2, pJAK2, CYLD, STAT3, pSTAT3, P62 and PDL1. (**M**) Schematic diagram showing the potential mechanism by which CAFs-secreted CXCL12 mediates PDL1 upregulation in bladder cancer

Protein ubiquitination is a dynamic process that is usually regulated by E3 ubiquitin ligases and DUBs [30]. Therefore, we next explored whether CXCL12 regulated the ubiquitination level of P62 by affecting some kind of DUB. Through intersection screening of several gene sets, only one DUB named CYLD was eventually obtained (Fig. 7A). Several other proven DUBs were also included in the study. Among them, USP15, ZRANB1, USP8, OTUD7B, and TRIM44 had been reported to be able to deubiquitinate P62 [31–35]. TNFAIP3 and JOSD1 were the intersection of the other three sets excluding the mass spectrometry results. The PCR results showed that the transcription of CYLD increased the most after CXCL12 stimulation, and a significant increase was already observed at 1 h (Fig. 7B). WB results also showed that CYLD presented a significant upregulation trend after CXCL12 stimulation, which was consistent with P62 and PDL1 (Fig. 7C; Figure S5A). AMD3100 could antagonize upregulation of CYLD by CXCL12 (Fig. 7D; Figure S5B). When CYLD was knocked down, the upregulation of P62 by CXCL12 was inhibited and the half-life of P62 was significantly shortened (Fig. 7E, F; Figure S5C, D). Therefore, we speculated that CYLD was the DUB of P62 in this progression. The IP results proved the protein interaction between CYLD and P62 (Fig. 7G; Figure S5E). The level of P62 ubiquitination significantly increased after CYLD knockdown (Fig. 7H, I). Next, we found that transfection of the CYLD plasmid in 293T cells effectively reduced ubiquitination of P62 (Fig. 7J).

CXCL12/CXCR4 axis activates various signaling pathways [36]. CXCL12 could promote rapid transcription of CYLD, so we further studied which pathway CXCL12 worked through and used P62 as the entry point. The TCGA database showed a positive correlation between P62 and the marker genes of the three pathways commonly activated by CXCL12 (Figure S6A, C, E). Stattic is the pSTAT3 inhibitor for JAK2/STAT3 pathway, MK2206 is the pAKT inhibitor for AKT/mTOR pathway and U0126 is the MEK inhibitor for ERK1/2 pathway. The three inhibitors were used to treat T24 cells and the results showed that only Sattic was able to downregulate P62. (Figure S6B, D, F). CYLD was also progressively downregulated with pSTAT3 inhibition, and both its downstream P62 and PDL1 were downregulated (Figure S6G). Inhibition of pSTAT3 with Stattic counteracted the elevation of CYLD by CXCL12 (Fig. 7K). Finally, CXCL12 time gradient stimulation demonstrated that CYLD as well as the downstream P62 and PDL1 were also upregulated with the activation of the JAK2/STAT3 pathway (Fig. 7L). Taken together, these results suggested that CXCL12 was able to activate the JAK2/STAT3 pathway, which promoted the transcription and expression of CYLD to regulate the P62 ubiquitination.

### Blocking CXCL12 inhibited tumor growth and promoted antitumor immunity

**Fig. 8:**
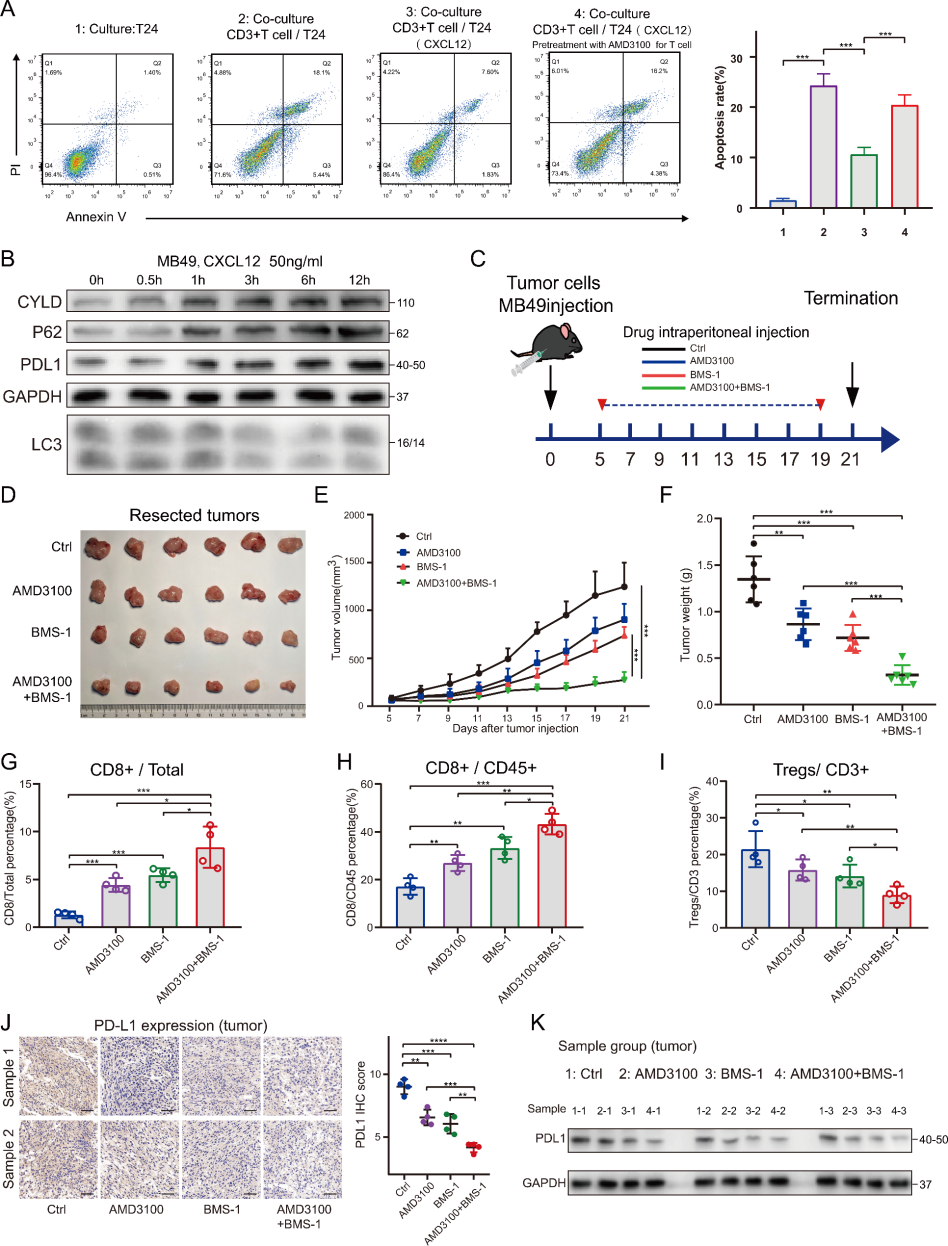
Blocking CXCL12 inhibited tumor growth and promoted antitumor immunity by decreasing PDL1 expression. (**A**) Left: Flow cytometry was used to determine the apoptosis rate of T24 cells after indirect co-culture with CD3 + T cells in the presence of CXCL12. Right: values were means ± SD from n = 3 independent experiments. (**B**) Western blotting showing the effect of murine CXCL12 on CYLD, P62, PDL1 and LC3 in MB49 cells. (**C**) Diagram of animal experiment procedure for combination therapy. (**D**) The images of resected tumors in each group. (**E**) The tumor growth curve of each group that received indicated treatment after inoculation of MB49 cells. Data were presented as means ± SD (n = 6). (**F**) Tumor weights of each group were shown as means ± SD (n = 6). (**G**) Flow cytometry analysis of CD8 + T cells percentage in total tumor cells of each group. Data were presented as means ± SD (n = 4). (**H**) Flow cytometry analysis of CD8 + T cells percentage in CD45 + T cells of each group. Data were presented as means ± SD (n = 4). (**I**) Flow cytometry analysis of regulatory cells (Tregs) percentage in CD3 + T cells of each group. Data were presented as means ± SD (n = 4). (**J**) Left: PDL1 expression in IHC of each group. Right: Quantitative IHC analysis of PDL1 staining (n = 4). (**K**) Western blotting showing the expression of PDL1 in tumor tissues of each group. * P < 0.05; ** P < 0.01; *** P < 0.001; **** P < 0.0001

T cell killing assay showed that CXCL12 was able to protect tumor cells from killing by T cells. The protective effect of CXCL12 against tumor was significantly attenuated when T cells were intervened with AMD3100 alone prior to co-culture. This suggested that CXCL12 could affect T cell function (Figure S7A; Fig. 8A). Next, we stimulated mouse bladder cancer cell MB49 with recombinant mouse CXCL12 prior to animal experiments. The results showed that CXCL12 could also cause upregulation of CYLD, P62 and PDL1 in MB49 cells (Fig. 8B).

The animal experimental protocol was shown in Fig. 8C. It was divided into four groups which were control group, AMD3100 group, BMS-1 group and co-medication group. BMS-1 is an inhibitor of the PD1/PDL1 protein/protein interaction and has been used in various tumor models [37, 38]. The results showed that both AMD3100 and BMS-1 alone could inhibit tumor growth, and the combination was more effective (Figure D-F). The screening process of CD4 + T cells, CD8 + T cells and FOXP3 + regulatory cells (Tregs) in mouse tumor tissues were shown in Figure S7B, C. The result of flow cytometry showed that AMD3100 was able to increase the number and proportion of CD8 + T cells and decrease the proportion of Tregs in TME. The combination of AMD3100 and BMS-1 could more effectively improve immune infiltration (Fig. 8G-I). The IHC and WB results also showed that AMD3100 combined with PD1/PDL1 inhibitor was able to downregulate PDL1 more significantly (Fig. 8J, K).

## Discussion

Our work revealed the prognostic value of CAFs in bladder cancer and the positive regulation of PDL1 by CAFs-derived CXCL12. In recent years, CAFs have gained prominence as a vital cell type influencing bladder cancer prognosis and curative effect [39]. With the development of scRNAseq technology, the heterogeneity of CAFs in different cancers has been gradually uncovered. CAFs originate from a variety of cell types and are characterized by increased expression of markers such as α-SMA, FAP, FSP1, PDGFR, and vimentin [40]. The CAFs that we extracted from bladder cancer tissue also showed a significant increase of α-SMA and FAP. CAFs have extremely strong pro-tumorigenic functions. On the one hand, as a component of tumor stroma, CAFs form a permeable barrier by remodeling the ECM to reduce drug efficacy. On the other hand, CAFs promote the expression of immune checkpoint molecules by secreting various cytokines and products, which indirectly affect the recruitment and activity of immune cells [41]. We demonstrated that CXCL12 was a key prognostic cytokine secreted mainly by CAFs in TME of bladder cancer. Both in vivo and in vitro experiments confirmed that CXCL12 could promote the proliferation, invasion and migration of bladder cancer cells, which is consistent with the findings of previous studies. Wang et al. revealed that CAFs-derived CXCL12 induced epithelial-mesenchymal transition of lung adenocarcinoma via CXCL12/β-catenin/PPARδ signaling [42]. Wei et al. reported that the CAFs in pancreatic cancer promoted progression and gemcitabine resistance via the CXCL12/SATB1 axis [43].

CXCL12 (also known as Stromal Cell-Derived Factor-1, SDF1) is a homeostatic chemokine widely expressed in different tissues. The role of CXCL12 in the pathogenesis of bladder cancer has been widely reported [44]. In the TME, the binding of CXCL12 to its major ligand CXCR4 initiates divergent signaling pathways downstream, which can result in a variety of responses such as chemotaxis, cell survival or proliferation and immune escape [45]. Lv et al. found that CXCL12 was associated with bladder cancer survival by integrating the TCGA database [46]. Wang et al. also revealed that CXCL12 may be involved in immune-related activities in bladder cancer through close crosstalk with immune microenvironment [47]. In this study, we highlight a novel role of CXCL12 in the regulation of PDL1 in bladder cancer. CXCL12 could rapidly up-regulate PDL1 of cancer cells in a short time. When CXCL12 of CAFs was silenced, the effect of CAFs on PDL1 was significantly weakened. Consistent with the findings of others, targeting CXCR4 in animal studies was able to reduce PDL1 infiltration in tumors [48, 49]. Moreover, our data emphasized that CXCL12 positively modulated PDL1 at the protein level rather than the mRNA level. We further found that CXCL12 was able to reduce the degradation of PDL1 by inhibiting autophagy but not the ubiquitination pathway. A study in stomach cancer showed a similar phenomenon that inhibition of autophagy by pharmacological inhibitors or small interfering RNAs increased the levels of PDL1 [50]. In another study, HIP1R deficiency in tumor cells led to the inhibition of selective autophagy degradation of PDL1, which in turn promoted immune escape [51].

Autophagy is a highly conserved lysosome-mediated degradation mechanism that can provide a continuous source of biomolecules and energy for cells to maintain homeostasis under stressful conditions such as the TME. Selective autophagy is mediated by autophagy receptors. Of these, P62 was the first to be identified and the most widely studied, and is closely associated with tumor development [29]. Li et al. reported that P62 promoted tumor growth by activating KEAP1/NRF2-dependent antioxidative response and could serve as a potential therapeutic target in bladder cancer [52]. Another team discovered a novel mechanism by which Sunitinib reduced PDL1 by promoting PDL1 degradation through P62-dependent selective autophagy. Sunitinib treatment promoted PDL1-P62 interactions, which may subsequently induce the degradation of PDL1 in autophagosomes and lysosomes [53]. In our study, after silencing P62, PDL1 was slightly upregulated and CXCL12 lost its upregulating effect on PDL1. After CXCL12 stimulation, the binding between PDL1 and P62 was reduced. Therefore, we inferred that P62 mediated the regulation of CXCL12 on PDL1 autophagy. In addition, the accumulation of P62 has been reported to have a direct effect on autophagy. Zhang et al. found that TSPO mediated P62 accumulation and autophagy Inhibition via interaction with P62, thereby promoting the increase of PDL1 [54]. The ubiquitination modification of P62 has been demonstrated to modulate its function. USP8 was reported to deubiquitinate P62 directly at the K420 site of UBA domain and inhibit P62-dependent autophagic activity [33]. Our results suggested that CXCL12 mediated the inhibition of P62 ubiquitination and identified CYLD as a deubiquitinase regulated by CXCL12 for P62.

CAFs-derived CXCL12 has been proven to be one of the most powerful chemokines regulating immune cells. As reported by Ene-Obong et al., CAFs were capable of reducing the migration of CD8 + T cells to juxtatumoral stromal compartment in a CXCL12-dependent manner, thereby reducing CD8 + T cell infiltration around tumor cells [55]. Another preclinical study revealed that the CXCL12 from FAP + CAFs resulted in the no-response of mice to immunological checkpoint antagonists α-CTLA4 and α-PDL1. The depletion of the FAP + stromal cells or inhibition of the receptor CXCR4 using AMD3100 could lead to rapid accumulation of CD8 + T cells and retard tumor growth [56]. In our study, treatment with AMD3100 improved the immune microenvironment, increasing the infiltration of CD8 + T cells and reducing the proportion of FOXP3 + Tregs in mouse tumors. In addition, the combination with PDL1/PDL1 inhibitor is more effective. These data suggest that blocking CXCL12 in the TME is a promising approach to improving the efficacy of immunotherapy.

## Conclusion

In summary, our study identified CXCL12 as a CAFs-derived cytokine with prognostic value and revealed a novel mechanism whereby CXCL12 inhibits P62-dependent autophagic degradation of PDL1 (Fig. 7M). In-depth study of CAFs will further elucidate the pathogenesis of bladder cancer, and targeting CAFs-derived CXCL12 may provide a potential therapeutic strategy for bladder cancer.

### Electronic supplementary material

Below is the link to the electronic supplementary material.

**Supplementary Material 1: Figure S1.** Prognostic value of CAFs and CXCL12 in TCGA and GEO databases. (**A**) Immunohistochemistry of α-SMA in bladder cancer tissues and normal tissues of HPA database. (**B**) Comparison of overall survival between high and low α-SMA groups. (**C**, **D**) Comparison of overall survival between high and low CAFs proportion groups, calculated by XCELL or MCPCOUNTER algorithm. (**E**, **F**) Comparison of overall survival between high and low CAFs proportion groups of another two GEO datasets. (**G**) Dot plots showing average expression of known markers in indicated cell clusters of scRNA-seq data. The dot size represents percent of cells expressing the genes in each cluster. The expression intensity of markers is shown. (**H**) Correlation between α-SMA and CXCL12. (**I**–**K**) Comparison of overall survival between high and low CXCL12 groups in TCGA or GEO database.

**Supplementary Material 2: Figure S2.** Effect of CXCL12 on proliferation, migration and invasion of UMUC3 cell line. (**A**) Western blotting showing the effect of different concentrations of CXCL12 on PDL1 in T24 cells. (**B**) The IC50 curves of AMD3100 in T24 and UMUC3 cell lines. The culture time was 48 h. (**C**) After the UMUC3 cells had been treated with PBS, CXCL12 (50 ng/ml), AMD3100 (10 µg/ml), or AMD3100 + CXCL12 for 24, 48 and 72 h, the cell viability was assessed using MTT assay. (**D**) Wound-healing assay and (**E**) transwell invasion assay were performed following treatment with PBS, CXCL12 (50 ng/ml), AMD3100 (10 µg/ml), or AMD3100 + CXCL12 for 24 h in UMUC3 cells. The statistical results were from three independent experiments. Values are means with SD. Scale bar = 300 μm.

**Supplementary Material 3: Figure S3.** Inhibition of autophagy degradation of PDL1 by CXCL12 in UMUC3 cell line. (**A**, **B**) The effect of CXCL12 (50 ng/ml) on PDL1 and P62 with or without siCXCR4 or AMD3100 (10 µg/ml), detected by western blotting in UMUC3 cells. (**C**) The expression of PDL1 within 0–24 h after CXCL12 stimulation detected by qPCR in UMUC3 cells. (**D**) Left: The degradation rate of PDL1 detected at the indicated time points by CHX chase assay (20µM) in UMUC3 cells treated with or without CXCL12 (50 ng/ml) stimulation. Right: Quantitative curve of the level of remained PDL1. (**E**) Western blotting of PDL1 ubiquitination stimulated by CXCL12 in UMUC3 cells. (**F**) Detection of the effect of CXCL12 on autophagy and PDL1 in UMUC3 cells by western blotting with PD-L1, P62 and LC3. (**G**) After inducing autophagy in UMUC3 cells using EBSS, the expression of PDL1 was detected by western blotting. (**H**) Immunofluorescence showing the UMUC3 cells that transfected EGFP-mCherry-LC3 plasmid co-incubated with CXCL12 (50ng/ml, 6 h). Scale bars = 20 μm. (**I**) The effect of chloroquine (10 µM, 12 h) or rapamycin (1 µM, 12 h) on PDL1 expression and autophagy with or without CXCL12 (50ng/ml) stimulation detected by western blotting in T24 cells. (**J**) Immunofluorescence showing the colocalization between PDL1 and LC3 after CXCL12 stimulation (10 µM, 12 h) in UMUC3 cells. (**K**) Western blotting showing the effect of knocking down ATG5 on PDL1 with or without CXCL12 (50 ng/ml, 6 h) stimulation in UMUC3 cells.

**Supplementary Material 4: Figure S4.** P62 mediated autophagic regulation of PDL1 by CXCL12 in UMUC3 cell line. (**A**) Detection of CXCL12 expression by qPCR after knocking down CXCL12 in CAFs. (**B**) Western blotting showing the effect of CAF-CM (12 h) on the expression of P62 and PDL in UMUC3 cells with or without AMD3100 pretreatment (10ug/ml,12 h). (**C**) After knockdown of CXCL12 in CAF, its culture medium (CM, 12 h) was used to stimulate to UMUC3 cells and then CXCL12 (50 ng/ml, 6 h) was added to rescue. Western blotting was used to detect changes of P62 and PDL1 expression. (**D**) Western blotting showing the effect of knocking down P62 on PDL1 expression with or without CXCL12 (50 ng/ml, 6 h) stimulation in UMUC3 cells. (**E**) Left: Compare the effect of CXCL12 (50 ng/ml, 0-12 h) on PDL1 and LC3 between the P62 knockdown and control groups of UMUC3 cells by western blotting. Right: Quantitative curves of protein changes of PDL1 and LC3. (**F**) Co-immunoprecipitation (co-IP) showing the physical interaction between PDL1 and P62 in UMUC3 cells. (**G**) Co-IP showing the effect of CXCL12 (50 ng/ml, 6 h) on the interaction between PDL1 and P62 in UMUC3 cells. (**H**) Left: Immunofluorescence showing the colocalization between PDL1 and P62 after stimulation with CXCL12 (50 ng/ml, 6 h) in UMUC3 cells. Scale bar = 10 μm. Right: The statistical results of colocalization factor (Pearson’s R value). Values are means ± SD from three independent experiments.

**Supplementary Material 5: Figure S5.** Regulation of CYLD by CXCL12 and effect of knockdown of CYLD on P62 in UMUC3 cell line. (**A**) UMUC3 cells were treated with CXCL12 for 0–12 h, detected by western blotting with CYLD, P62 and PDL1. (**B**) Western blotting showing the effect of CXCL12 (50ng/ml, 6 h) on CYLD, P62 and PDL1 in UMUC3 cells with or without AMD3100 pretreatment (10ug/ml,12 h). (**C**) Western blotting showing the effect of knocking down CYLD on P62 and PDL1 with or without CXCL12 (50ng/ml, 6 h) stimulation in UMUC3 cells. (**D**) Left: The degradation rate of P62 detected at the indicated time points by CHX chase assay (20µM) in UMUC3 cells interfered by siCYLD. Right: Quantitative curve of the level of remained P62. (**E**) Co-immunoprecipitation (co-IP) showing the physical interaction between CYLD and P62 in UMUC3 cells.

**Supplementary Material 6: Figure S6.** The effects of inhibitors of three signaling pathways on P62. (**A**) Correlation between JAK2/STAT3 and P62 in TCGA. (**B**) Western blotting showing the effect of pSTAT3 inhibitor Stattic (12 h) on P62 in T24 cells. (**C**) Correlation between AKT/MTOR and P62 in TCGA. (**D**) Western blotting showing the effect of pAKT inhibitor MK2206 (12 h) on P62 in T24 cells. (**E**) Correlation between Erk1/2 and P62 in TCGA. (**F**) Western blotting showing the effect of MEK1/2 inhibitor U0126 (12 h) on P62 in T24 cells. (**G**) Western blotting showing the effect of pSTAT3 inhibitor Stattic (0-12 h) on CYLD, P62 and PDL1 in T24 cells.

**Supplementary Material 7: Figure S7.** Identification of human peripheral blood CD3 + T cells (**A**) and screening process for CD4 + T cells, CD8 + T cells (**B**) and FOXP3 + Tregs (**C**) in mouse tumors.

**Supplementary Material 8: Table S1.** The antibodies and reagents used in this study.

**Supplementary Material 9: Table S2.** Sequences of all siRNAs and RNA primers used in this study.

## Data Availability

All remaining data and materials are available from the authors upon reasonable request.

## Abbreviations

CAFs: Cancer-associated fibroblasts
NFs: Normal fibroblasts
TME: Tumor microenvironment
ECM: Extracellular matrix
CXCL12: C-X-C motif chemokine 12
CXCR4: C-X-C chemokine receptor type 4
PDL1: Programmed cell death 1 ligand 1
P62: Sequestosome-1
CYLD: cylindromatosis
MIBC: muscle-invasive bladder cancer
NMIBC: non-muscle-invasive bladder cancer
TCGA: The Cancer Genome Atlas
KEGG: Kyoto Encyclopedia of Genes and Genomes
GSEA: Gene Set Enrichment Analysis
GSVA: Gene Set Variation Analysis
scRNA: single cell RNA
DUBs: deubiquitinases
LC-MS/MS: Liquid Chromatography-Mass Spectrometry/Mass Spectrometry
