# Supplementary figures and images for: Cancer-associated fibroblasts-derived CXCL12 enhances immune escape of bladder cancer through inhibiting P62-mediated autophagic degradation of PDL1
Source: J Exp Clin Cancer Res. 2023 Nov 25;42:316. doi: 10.1186/s13046-023-02900-0 (PMC10675892; doi:10.1186/s13046-023-02900-0)

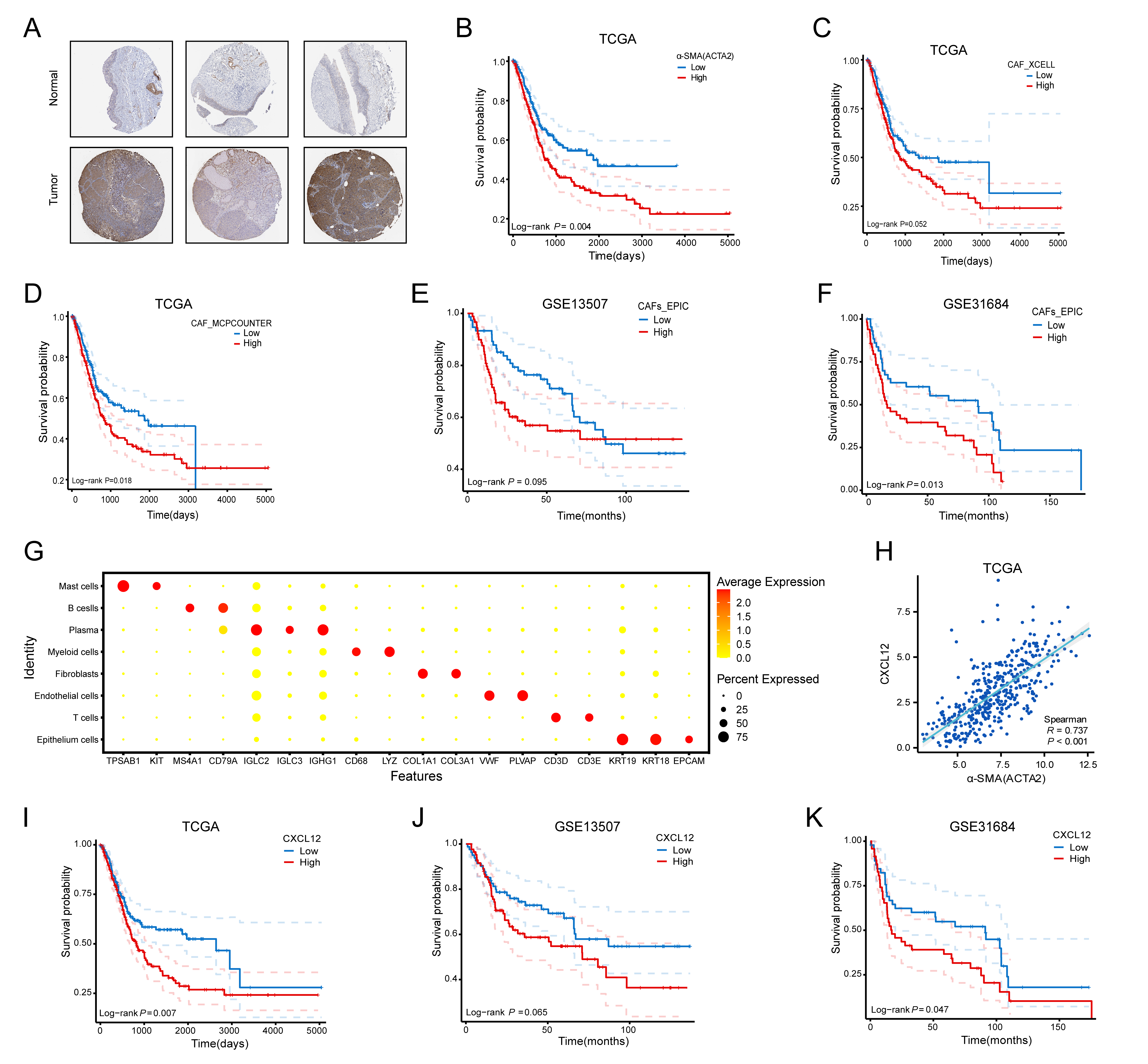

Supplement: Supplementary file 1 — Supplementary Material 1: Figure S1. Prognostic value of CAFs and CXCL12 in TCGA and GEO databases. (A) Immunohistochemistry of α-SMA in bladder cancer tissues and normal tissues of HPA database. (B) Comparison of overall survival between high and low α-SMA groups. (C, D) Comparison of overall survival between high and low CAFs proportion groups, calculated by XCELL or MCPCOUNTER algorithm. (E, F) Comparison of overall survival between high and low CAFs proportion groups of another two GEO datasets. (G) Dot plots showing average expression of known markers in indicated cell clusters of scRNA-seq data. The dot size represents percent of cells expressing the genes in each cluster. The expression intensity of markers is shown. (H) Correlation between α-SMA and CXCL12. (I–K) Comparison of overall survival between high and low CXCL12 groups in TCGA or GEO database. [file 13046_2023_2900_MOESM1_ESM.tif]

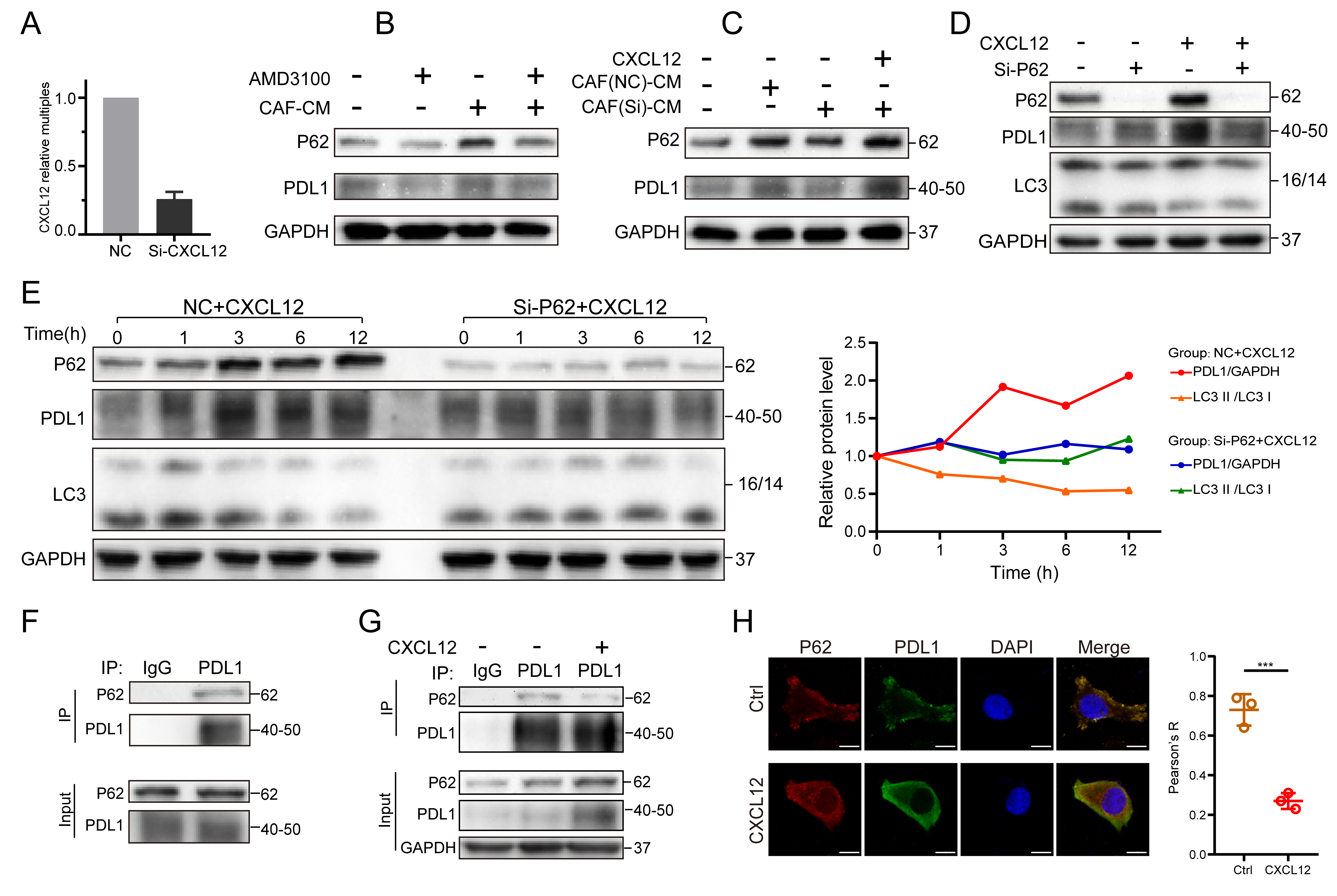

Supplement: Supplementary file 2 — Supplementary Material 2: Figure S2. Effect of CXCL12 on proliferation, migration and invasion of UMUC3 cell line. (A) Western blotting showing the effect of different concentrations of CXCL12 on PDL1 in T24 cells. (B) The IC50 curves of AMD3100 in T24 and UMUC3 cell lines. The culture time was 48 h. (C) After the UMUC3 cells had been treated with PBS, CXCL12 (50 ng/ml), AMD3100 (10 µg/ml), or AMD3100 + CXCL12 for 24, 48 and 72 h, the cell viability was assessed using MTT assay. (D) Wound-healing assay and (E) transwell invasion assay were performed following treatment with PBS, CXCL12 (50 ng/ml), AMD3100 (10 µg/ml), or AMD3100 + CXCL12 for 24 h in UMUC3 cells. The statistical results were from three independent experiments. Values are means with SD. Scale bar = 300 μm. [file 13046_2023_2900_MOESM2_ESM.tif]

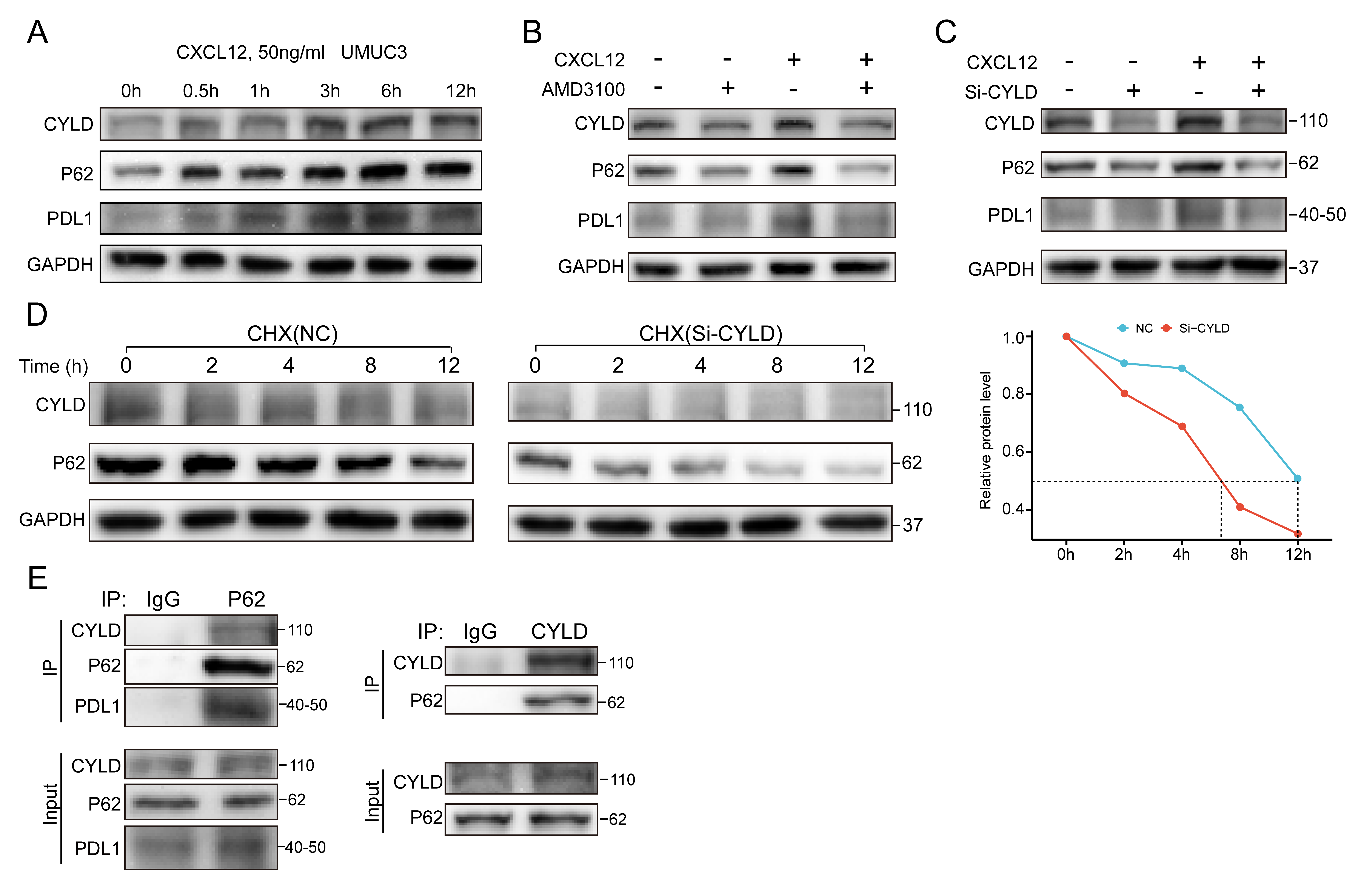

Supplement: Supplementary file 3 — Supplementary Material 3: Figure S3. Inhibition of autophagy degradation of PDL1 by CXCL12 in UMUC3 cell line. (A, B) The effect of CXCL12 (50 ng/ml) on PDL1 and P62 with or without siCXCR4 or AMD3100 (10 µg/ml), detected by western blotting in UMUC3 cells. (C) The expression of PDL1 within 0–24 h after CXCL12 stimulation detected by qPCR in UMUC3 cells. (D) Left: The degradation rate of PDL1 detected at the indicated time points by CHX chase assay (20µM) in UMUC3 cells treated with or without CXCL12 (50 ng/ml) stimulation. Right: Quantitative curve of the level of remained PDL1. (E) Western blotting of PDL1 ubiquitination stimulated by CXCL12 in UMUC3 cells. (F) Detection of the effect of CXCL12 on autophagy and PDL1 in UMUC3 cells by western blotting with PD-L1, P62 and LC3. (G) After inducing autophagy in UMUC3 cells using EBSS, the expression of PDL1 was detected by western blotting. (H) Immunofluorescence showing the UMUC3 cells that transfected EGFP-mCherry-LC3 plasmid co-incubated with CXCL12 (50ng/ml, 6 h). Scale bars = 20 μm. (I) The effect of chloroquine (10 µM, 12 h) or rapamycin (1 µM, 12 h) on PDL1 expression and autophagy with or without CXCL12 (50ng/ml) stimulation detected by western blotting in T24 cells. (J) Immunofluorescence showing the colocalization between PDL1 and LC3 after CXCL12 stimulation (10 µM, 12 h) in UMUC3 cells. (K) Western blotting showing the effect of knocking down ATG5 on PDL1 with or without CXCL12 (50 ng/ml, 6 h) stimulation in UMUC3 cells. [file 13046_2023_2900_MOESM3_ESM.tif]

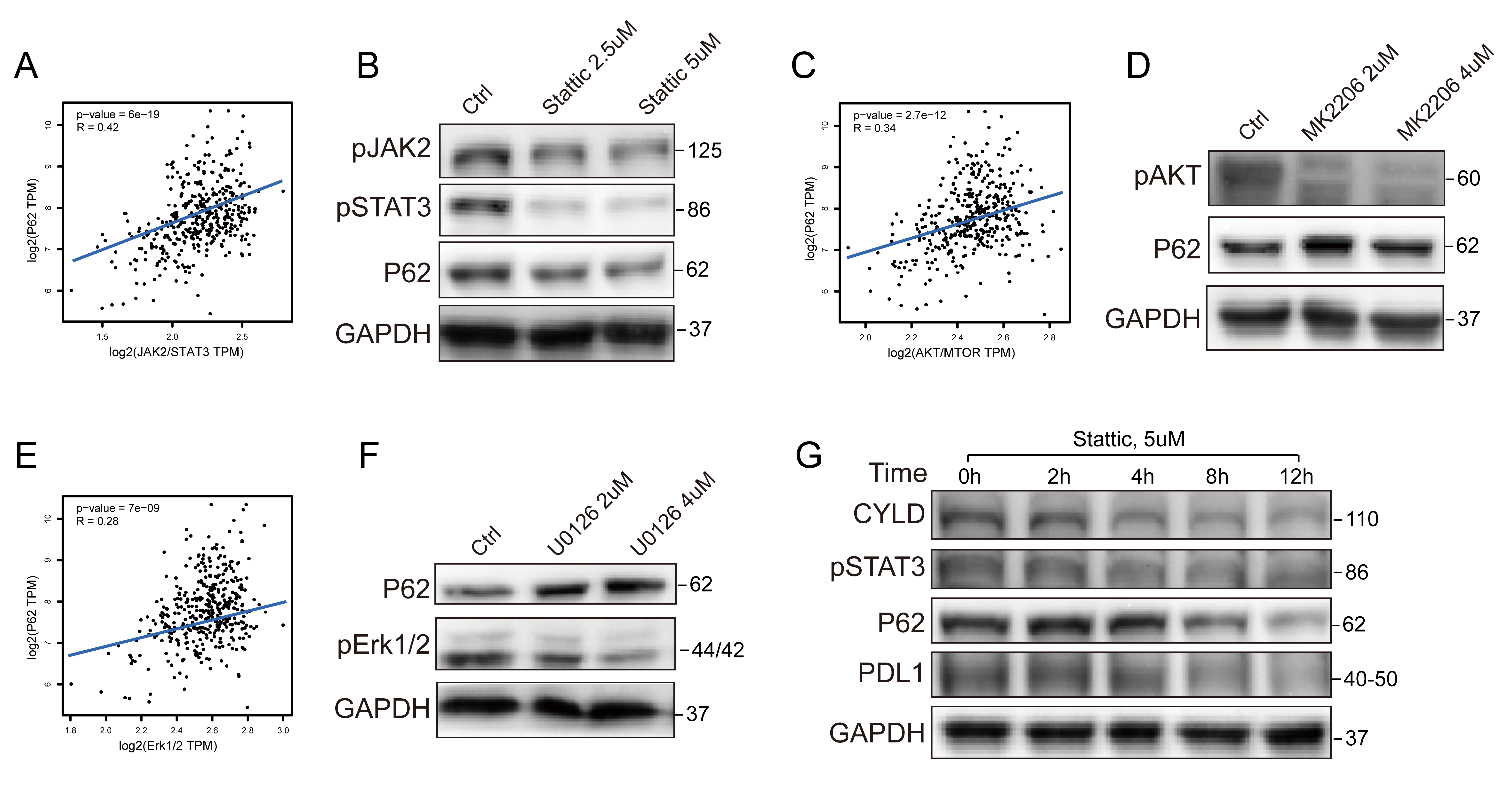

Supplement: Supplementary file 4 — Supplementary Material 4: Figure S4. P62 mediated autophagic regulation of PDL1 by CXCL12 in UMUC3 cell line. (A) Detection of CXCL12 expression by qPCR after knocking down CXCL12 in CAFs. (B) Western blotting showing the effect of CAF-CM (12 h) on the expression of P62 and PDL in UMUC3 cells with or without AMD3100 pretreatment (10ug/ml,12 h). (C) After knockdown of CXCL12 in CAF, its culture medium (CM, 12 h) was used to stimulate to UMUC3 cells and then CXCL12 (50 ng/ml, 6 h) was added to rescue. Western blotting was used to detect changes of P62 and PDL1 expression. (D) Western blotting showing the effect of knocking down P62 on PDL1 expression with or without CXCL12 (50 ng/ml, 6 h) stimulation in UMUC3 cells. (E) Left: Compare the effect of CXCL12 (50 ng/ml, 0-12 h) on PDL1 and LC3 between the P62 knockdown and control groups of UMUC3 cells by western blotting. Right: Quantitative curves of protein changes of PDL1 and LC3. (F) Co-immunoprecipitation (co-IP) showing the physical interaction between PDL1 and P62 in UMUC3 cells. (G) Co-IP showing the effect of CXCL12 (50 ng/ml, 6 h) on the interaction between PDL1 and P62 in UMUC3 cells. (H) Left: Immunofluorescence showing the colocalization between PDL1 and P62 after stimulation with CXCL12 (50 ng/ml, 6 h) in UMUC3 cells. Scale bar = 10 μm. Right: The statistical results of colocalization factor (Pearson’s R value). Values are means ± SD from three independent experiments. [file 13046_2023_2900_MOESM4_ESM.tif]

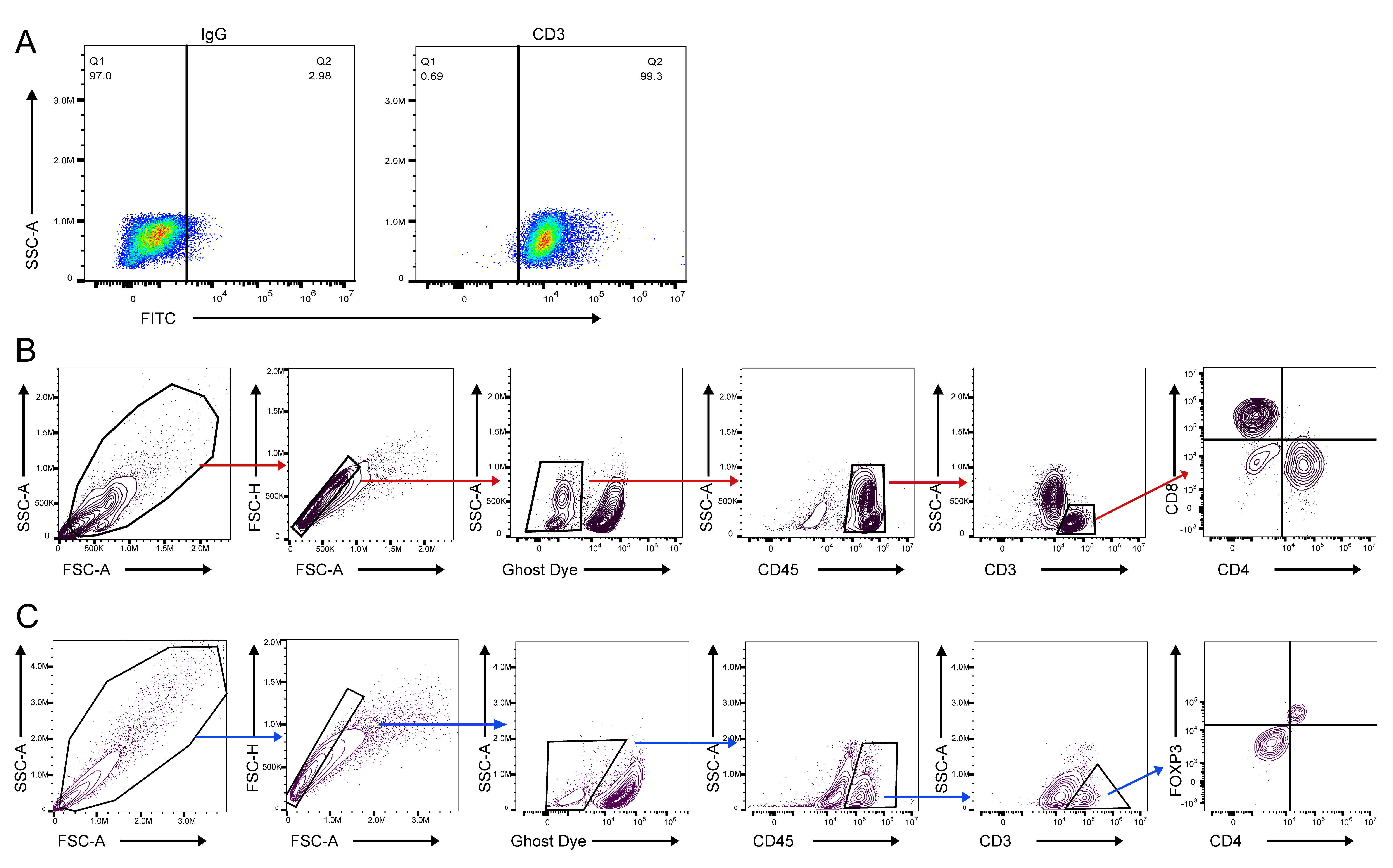

Supplement: Supplementary file 5 — Supplementary Material 5: Figure S5. Regulation of CYLD by CXCL12 and effect of knockdown of CYLD on P62 in UMUC3 cell line. (A) UMUC3 cells were treated with CXCL12 for 0–12 h, detected by western blotting with CYLD, P62 and PDL1. (B) Western blotting showing the effect of CXCL12 (50ng/ml, 6 h) on CYLD, P62 and PDL1 in UMUC3 cells with or without AMD3100 pretreatment (10ug/ml,12 h). (C) Western blotting showing the effect of knocking down CYLD on P62 and PDL1 with or without CXCL12 (50ng/ml, 6 h) stimulation in UMUC3 cells. (D) Left: The degradation rate of P62 detected at the indicated time points by CHX chase assay (20µM) in UMUC3 cells interfered by siCYLD. Right: Quantitative curve of the level of remained P62. (E) Co-immunoprecipitation (co-IP) showing the physical interaction between CYLD and P62 in UMUC3 cells. [file 13046_2023_2900_MOESM5_ESM.tif]

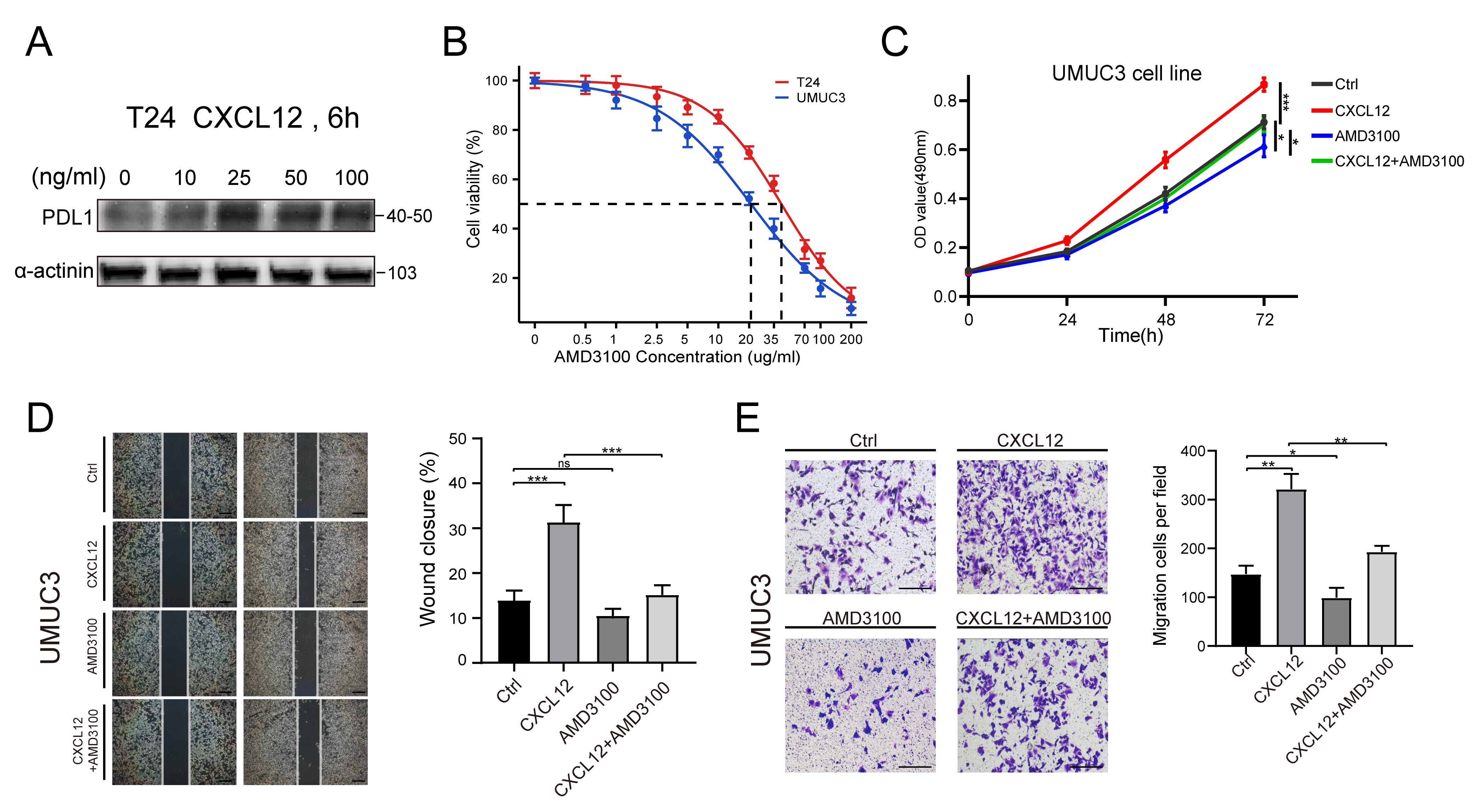

Supplement: Supplementary file 6 — Supplementary Material 6: Figure S6. The effects of inhibitors of three signaling pathways on P62. (A) Correlation between JAK2/STAT3 and P62 in TCGA. (B) Western blotting showing the effect of pSTAT3 inhibitor Stattic (12 h) on P62 in T24 cells. (C) Correlation between AKT/MTOR and P62 in TCGA. (D) Western blotting showing the effect of pAKT inhibitor MK2206 (12 h) on P62 in T24 cells. (E) Correlation between Erk1/2 and P62 in TCGA. (F) Western blotting showing the effect of MEK1/2 inhibitor U0126 (12 h) on P62 in T24 cells. (G) Western blotting showing the effect of pSTAT3 inhibitor Stattic (0-12 h) on CYLD, P62 and PDL1 in T24 cells. [file 13046_2023_2900_MOESM6_ESM.tif]

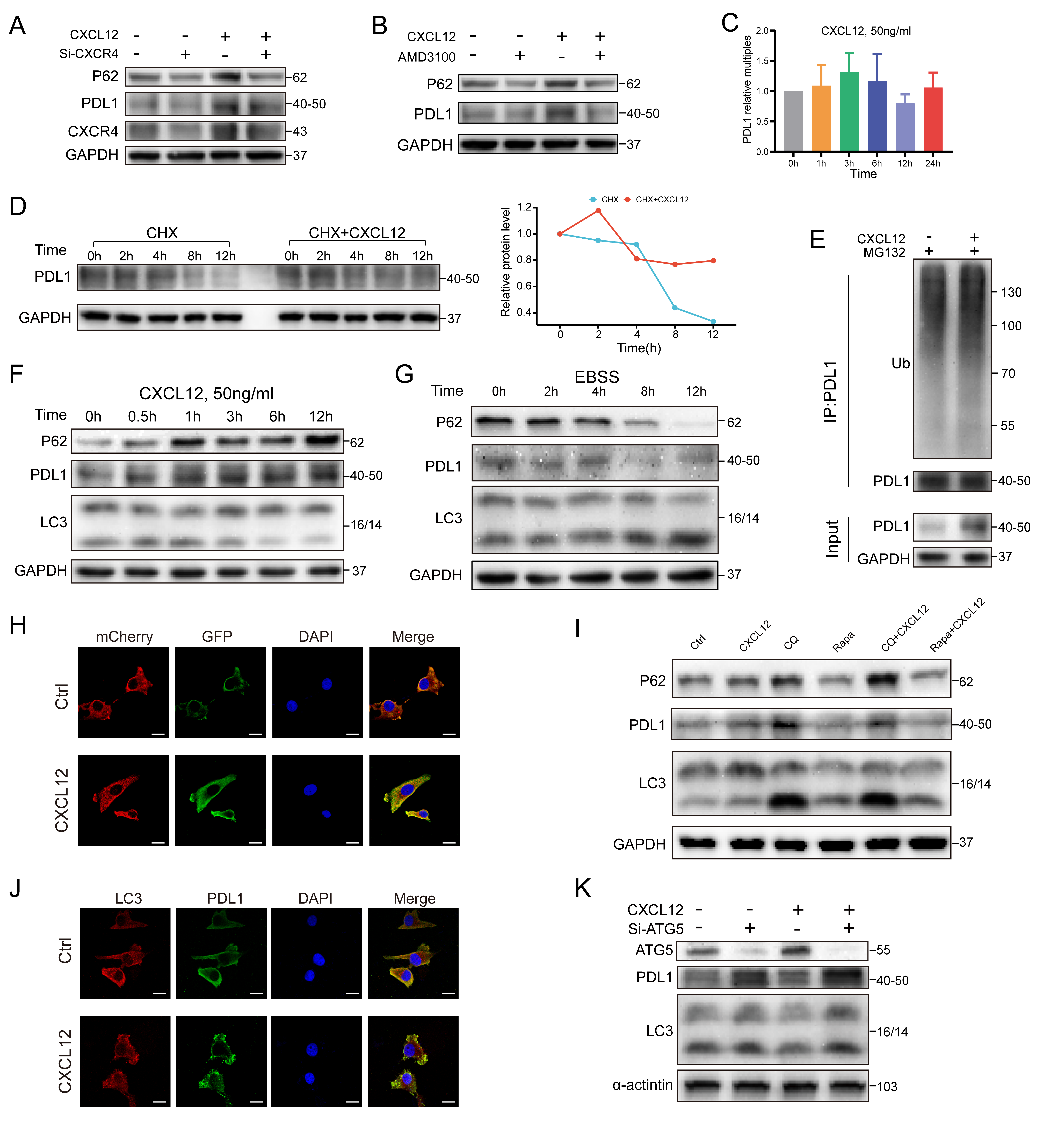

Supplement: Supplementary file 7 — Supplementary Material 7: Figure S7. Identification of human peripheral blood CD3 + T cells (A) and screening process for CD4 + T cells, CD8 + T cells (B) and FOXP3 + Tregs (C) in mouse tumors. [file 13046_2023_2900_MOESM7_ESM.tif]
