# Supplementary material for: Cancer-associated fibroblasts-derived CXCL12 enhances immune escape of bladder cancer through inhibiting P62-mediated autophagic degradation of PDL1
Source: J Exp Clin Cancer Res. 2023 Nov 25;42:316. doi: 10.1186/s13046-023-02900-0 (PMC10675892; doi:10.1186/s13046-023-02900-0)
Supplement: Supplementary file 8 — Supplementary Material 8: Table S1. The antibodies and reagents used in this study. [file 13046_2023_2900_MOESM8_ESM.docx]

**Table S1. Antibodies and Reagents**

| **Antibodies** | **Source** | **Identifier** | **Application** |
| --- | --- | --- | --- |
| Rabbit anti-FAP | Cell Signaling Technology, MA, USA | Cat#66562, Lot#13 | WB (human cells) |
| Rabbit anti-α-SMA | Proteintech, Rosemont, IL, USA | Cat#55135-1-AP, Lot#00109134 | WB, IF, IHC (human cells) |
| Rabbit anti-CXCL12 | Proteintech, Rosemont, IL, USA | Cat# 17402-1-AP, Lot#00048529 | IHC (human cells) |
| Rabbit anti-Vimentin | Cell Signaling Technology, MA, USA | Cat#5741, Lot#5 | WB (human cells) |
| Rabbit anti-PDL1 | Cell Signaling Technology, MA, USA | Cat#13684, Lot#18 | WB (human cells) |
| Rabbit anti-PDL1 | Proteintech, Rosemont, IL, USA | Cat#28076-1-AP, Lot#00079254 | WB, IF, IHC (human cells, mouse cells) |
| Mouse anti-P62 | Cell Signaling Technology, MA, USA | Cat#88588, Lot#3 | WB, IF (human cells) |
| Rabbit anti-LC3B | Cell Signaling Technology, MA, USA | Cat#2775, Lot#13 | WB, IF (human cells) |
| Rabbit anti-ATG5 | Abmart, Shanghai, China | Cat#T55766, Lot#10044603 | WB (human cells) |
| Mouse anti-Ubiquitin | Cell Signaling Technology, MA, USA | Cat#3936, Lot#19 | WB (human cells) |
| Rabbit anti-CYLD | Proteintech, Rosemont, IL, USA | Cat#11110-1-AP, Lot#00080198 | WB (human cells) |
| Rabbit anti-HA-Tag | Cell Signaling Technology, MA, USA | Cat#3724, Lot#16 | WB (human cells) |
| Mouse anti-Flag-Tag | Abmart, Shanghai, China | Cat#M20008, Lot#10042990 | WB (human cells) |
| Rabbit anti-pAKT | Cell Signaling Technology, MA, USA | Cat#4060, Lot#26 | WB (human cells) |
| Rabbit anti-pERK | Cell Signaling Technology, MA, USA | Cat#4695, Lot#21 | WB (human cells) |
| Rabbit anti-pJAK2 | Cell Signaling Technology, MA, USA | Cat#3776, Lot#13 | WB (human cells) |
| Rabbit anti-JAK2 | Cell Signaling Technology, MA, USA | Cat#3230, Lot#13 | WB (human cells) |
| Rabbit anti-pSTAT3 | Cell Signaling Technology, MA, USA | Cat#9145, Lot#43 | WB (human cells) |
| Mouse anti-STAT3 | Cell Signaling Technology, MA, USA | Cat#9139, Lot#16 | WB (human cells) |
| Rabbit anti-α-actintin | Proteintech, Rosemont, IL, USA | Cat#11313-2-AP, Lot#00082167 | WB (human cells) |
| Rabbit anti-GAPDH | Cell Signaling Technology, MA, USA | Cat#5174, Lot#16 | WB (human cells, mouse cells) |
| FITC anti-human CD3 | Invitrogen, Carlsbad, California, USA | Cat#11-0039-42, Lot#4330459 | FCM (human cells) |
| PerCP/Cyanine5.5 Anti-Mouse CD45 | Elabscience, Wuhan, China | Cat#E-AB-F1136J, Lot#AF13373 | FCM (mouse cells) |
| APC anti-Mouse CD3 | Elabscience, Wuhan, China | Cat#E-AB-F1013E, Lot#AF11661 | FCM (mouse cells) |
| PE anti-Mouse CD3 | Elabscience, Wuhan, China | Cat#E-AB-F1013D, Lot#AF11660 | FCM (mouse cells) |
| FITC anti-Mouse CD4 | Elabscience, Wuhan, China | Cat#E-AB-F1097C, Lot#AF11650 | FCM (mouse cells) |
| PE anti-Mouse CD8a | Elabscience, Wuhan, China | Cat#E-AB-F1104D, Lot#AF11951 | FCM (mouse cells) |
| APC anti-Mouse Foxp3 | Elabscience, Wuhan, China | Cat#E-AB-F1238E, Lot#AF10938 | FCM (mouse cells) |
| Anti-Mouse CD16/32 | Elabscience, Wuhan, China | Cat#E-AB-F0997A, Lot#220210 | FCM (mouse cells) |

WB: western blotting; IF: immunofluorescence; IHC: immunohistochemistry; FCM: flow cytometry.

| **Reagents** | **Source** | **Identifier** |
| --- | --- | --- |
| Collagenase Ⅰ | Solarbio Life science, Beijing, China | Cat#C8140 |
| Hyaluronidase | Solarbio Life science, Beijing, China | Cat#H8030 |
| recombinant human SDF-1α/CXCL12α | Sangon Biotech, Shanghai, China | Cat#C600189 |
| recombinant murine SDF-1α/CXCL12α | Sangon Biotech, Shanghai, China | Cat#C600190 |
| AMD3100 | MedChemExpress, Monmouth Junction, NJ, USA | Cat#HY-10046 |
| MG132 | MedChemExpress, Monmouth Junction, NJ, USA | Cat#HY-13259 |
| Cycloheximide | MedChemExpress, Monmouth Junction, NJ, USA | Cat#HY-12320 |
| Chloroquine | MedChemExpress, Monmouth Junction, NJ, USA | Cat#HY-17589A |
| Rapamycin | MedChemExpress, Monmouth Junction, NJ, USA | Cat#HY-10219 |
| BMS-1 | MedChemExpress, Monmouth Junction, NJ, USA | Cat#HY-19991 |
| DAPI solution | Solarbio Life science, Beijing, China | Cat#C0065 |
| Human lymphocyte separation medium | Solarbio Life science, Beijing, China | Cat#P8610 |
| Ghost Dye™ Violet 510 | Tonbo Biosciences, Santiago, USA | Cat#13-0870-T100 |
