# Supplementary material for: Cancer-associated fibroblasts-derived CXCL12 enhances immune escape of bladder cancer through inhibiting P62-mediated autophagic degradation of PDL1
Source: J Exp Clin Cancer Res. 2023 Nov 25;42:316. doi: 10.1186/s13046-023-02900-0 (PMC10675892; doi:10.1186/s13046-023-02900-0)
Supplement: Supplementary file 9 — Supplementary Material 9: Table S2. Sequences of all siRNAs and RNA primers used in this study. [file 13046_2023_2900_MOESM9_ESM.docx]

**Table S2. Sequences of all siRNAs and RNA primers used in this study.**

Small interfering RNA

| Gene | Primer | Sequence |
| --- | --- | --- |
| CXCR4 | Sense | CUGUCCUGCUAUUGCAUUATT |
|  | Antisense | UAAUGCAAUAGCAGGACAGTT |
| P62 | Sense | GACAUCUUCCGAAUCUACATT |
|  | Antisense | UGUAGAUUCGGAAGAUGUCTT |
| ATG5 | Sense | GACGUUGGUAACUGACAAATT |
|  | Antisense | UUUGUCAGUUACCAACGUCTT |
| CXCL12 | Sense | CGUCAAGCAUCUCAAAAUUTT |
|  | Antisense | AAUUUUGAGAUGCUUGACGTT |
| CYLD | Sense | GUAUAGGACAGUAUAUUCATT |
|  | Antisense | UGAAUAUACUGUCCUAUACTT |

All primers for qRT-PCR

| Gene | Primer | Sequence (5' -> 3') |
| --- | --- | --- |
| PDL1 | Forward | TATGGTGGTGCCGACTACAA |
|  | Reverse | TGCTTGTCCAGATGACTTCG |
| P62 | Forward | AGAACGTTGGGGAGAGTGTG |
|  | Reverse | GCGATCTTCCTCATCTGCTC |
| CXCL12 | Forward | AACACTCCAAACTGTGCCCT |
|  | Reverse | CTCTCACATCTTGAACCTCTTGTT |
| USP20 | Forward | CAATGGGCAGTGGTACGAGT |
|  | Reverse | CCGCGAAGGTGTTGAACTTG |
| ZRANB1 | Forward | ACAGCCTGCATGACTGTTCA |
|  | Reverse | CCCAGTCTTCTTGCCACTGT |
| USP8 | Forward | ACAGCCTGCATGACTGTTCA |
|  | Reverse | TGGGCAGCAGGTTTAGAAGG |
| OTUD7B | Forward | GTCAGATTTTGTCCGTTCCACA |
|  | Reverse | CATGGACTTGACGTAGCTGTT |
| TRIM44 | Forward | AGGCAGCTCATCTGTGTCCT |
|  | Reverse | GCCTTCAGTCCACCTGAGTC |
| TNFAIP3 | Forward | AGAGCAACTGAGATCGAGCCA |
|  | Reverse | CTGGTTGGGATGCTGACACTC |
| JOSD1 | Forward | GGGATACGCTGCAAGAGATTT |
|  | Reverse | CCATGACGTTAGTGAGGGCA |
| CYLD | Forward | TCTATGGGGTAATCCGTTGG |
|  | Reverse | CAGCCTGCACACTCATCTTC |
| β-actin | Forward | GGCATCGTCACCAACTGGGAC |
|  | Reverse | CGATTTCCCGCTCGGCCGTGG |
